# Comprehensive analysis of cuproptosis-related lncRNAs for prognostic significance and immune microenvironment characterization in hepatocellular carcinoma

**DOI:** 10.3389/fimmu.2022.991604

**Published:** 2023-01-04

**Authors:** Duguang Li, Shengxi Jin, Peng Chen, Yiyin Zhang, Yirun Li, Cheng Zhong, Xiaoxiao Fan, Hui Lin

**Affiliations:** ^1^ Department of General Surgery, Sir Run Run Shaw Hospital, School of Medicine, Zhejiang University, Hangzhou, China; ^2^ Zhejiang Engineering Research Center of Cognitive Healthcare, Sir Run Run Shaw Hospital, School of Medicine, Zhejiang University, Hangzhou, China; ^3^ College of Biomedical Engineering and Instrument Science, Zhejiang University, Hangzhou, China

**Keywords:** lncRNA, hepatocellular carcinoma, immune microenvironment, survival analysis, cuproptosis

## Abstract

Cuproptosis was characterized as a novel type of programmed cell death. Recently, however, the role of cuproptosis-related long noncoding RNAs (CRLs) in tumors has not yet been studied. Identifying a predictive CRL signature in hepatocellular carcinoma (HCC) and investigating its putative molecular function were the goals of this work. Initially, Pearson’s test was used to assess the relationship between lncRNAs and cuproptosis-associated genes obtained from HCC data of The Cancer Genome Atlas (TCGA). By implementing differential expression and univariate Cox analysis, 61 prognostic CRLs were subsequent to the least absolute shrinkage and selection operator (LASSO) Cox regression analysis. A prognostic risk score model was then constructed to evaluate its ability to predict patients’ survival when combined with clinicopathological parameters in HCC. The five-lncRNA prognostic signature categorized the HCC patients into high- and low-risk groups. The low-risk group exhibited more sensitivity to elesclomol than the high-risk one. Surprisingly, distinct mitochondrial metabolism pathways connected to cuproptosis and pivotal immune-related pathways were observed between the two groups *via* gene set enrichment analysis (GSEA). Meanwhile, there were substantial differences between the high-risk group and the low-risk group in terms of tumor-infiltrating immune cells (TIICs). Furthermore, a positive relationship was shown between the risk score and the expression of immune checkpoints. Additionally, differential expression of the five lncRNAs was confirmed in our own HCC samples and cell lines *via* RT-qPCR. Finally, *in vitro* assays confirmed that WARS2-AS1 and MKLN1-AS knockdown could sensitize HCC cells to elesclomol-induced cuproptosis. Overall, our predictive signature may predict the prognosis of HCC patients in an independent manner, give a better understanding of how CRLs work in HCC, and offer therapeutic reference for patients with HCC.

## Introduction

Hepatocellular carcinoma remains one of the most common malignancies globally, ranking as the third cause of cancer-related mortality and sixth with regard to the incidence of all tumor types (1). A number of risk factors for the occurrence of HCC include hepatitis B virus (HBV), hepatitis C virus (HCV), alcoholic liver disease, and nonalcoholic steatohepatitis (2). Due to the low rate of early diagnosis in HCC, the majority of cases are not detected until they have progressed to an advanced stage of cancer (3). Over the past decades, many different treatment modalities have been developed to combat HCC, but high post-operative recurrence rates and drug resistance continue to be barriers to cure HCC (4, 5). As a result, a thorough understanding of the networks involved in HCC development and progression is essential for improving detection efficiency and developing more effective therapies.

It is known that copper (Cu) is a cofactor for enzymes that control a variety of vital cellular activities, including mitochondrial respiration, antioxidant defense, and the manufacture of hormones (6). According to recent findings, although Cu is increasingly implicated in cell proliferation (7), dysregulation of Cu stores can also induce cytotoxicity *via* multiple pathways. One mechanism proposed by Masazumi Nagai et al. demonstrated that the Cu-binding drug elesclomol preferentially chelated Cu outside of cells and selectively transported the Cu to mitochondria as elesclomol-Cu (II), thereby triggering mitochondrial reactive oxygen species (ROS) induction (8). Another Cu chelators disulfiram (DSF) formed a DSF/Cu complex with Cu to severely disrupt mitochondrial homeostasis and increase the free iron pool, ultimately provoking lipid peroxidation and causing ferroptotic cell death (9). However, these traditional views of copper-induced cytotoxicity have been challenged by emerging evidence that Cu-dependent death occurs *via* direct binding of Cu to lipoylated components of the TCA cycle, which leads to proteotoxic stress and ultimately cell death (10). In addition, treatment with inhibitors of other well-known cell death pathways, such as apoptosis (Z-VAD-FMK), ferroptosis (ferrostatin-1), necroptosis (necrostatin-1), and oxidative stress (N-acetyl cysteine), could not reverse Cu ionophore-induced cell death. Therefore, scientists proposed this previously uncharacterized cell death mechanism as cuproptosis. So far, there have only been a few cuproptosis-related genes discovered in cancer. It is urgent for us to find novel regulators of cuproptosis for the purpose of improving the diagnosis and treatment of cancer.

In recent years, advances in sequencing technologies have led to the discovery of a multitude of non-coding RNA (ncRNA) species, which are a class of RNAs lacking potential to encode proteins. Pervasive transcription produces a vast repertoire of ncRNAs of all sizes and shapes, including short ncRNAs (such as microRNAs), long non-coding RNAs (lncRNAs), and circular RNAs (cirRNAs) (11). ncRNA containing more than 200 nucleotides was defined as lncRNA, which can act as miRNA sponges, RNA-binding protein sequestering factors, as well as regulators of gene expression by controlling mRNA transcription (12). LncRNAs have been implicated in tumorigenesis-associated biological functions such as metastasis and programmed death, according to a growing body of research over the last several decades (13, 14). Despite the fact that lncRNAs have been studied extensively in other types of cell death (15), no study on cuproptosis-associated lncRNAs has yet been documented. It is thus a great challenge for us to explore how lncRNAs work during the process of cuproptosis especially in cancer. Intriguingly, studies have also shown that cell death-related lncRNA could exert a pivotal role in the regulation of immune cells. For example, three lncRNA A2M-AS1, C2orf27A, and ZNF667-AS1 were identified as the upstream transcriptional regulators of several hub ferroptosis-associated genes (FAGs) while these FAGs had a significant effect on immune cell infiltration in gastric cancer, indicating that lncRNA might affect immune response *via* mediating ferroptosis process (16). Moreover, another literature has reported that the construction of a ferroptosis-related lncRNA model could contribute to the immune status and response to immunotherapy of lung cancer, which built a link between cell death-related lncRNAs and immune regulation (17). Such findings have provided us with new perspectives on the investigation of the relationship between the cuproptosis-related lncRNAs and tumor-associated pathways in the future.

In this work, we developed a predictive model based on cuproptosis-associated lncRNAs in HCC. The performance of predicting survival on the basis of the signature-based risk score was analyzed together with other standard clinicopathological parameters. In addition, its significance in assessing the sensitivity of cuproptosis inducer elesclomol has been further evaluated between the high- and low- risk groups. Moreover, internal cohorts were then carried out to verify the results above. Further investigation into the mechanism of action of CRLs in HCC was conducted using gene set enrichment analysis (GSEA). and immune infiltration analysis. Finally, the role of three lncRNAs in regulating cuproptosis was validated *via in vitro* experiments. Overall, our findings provided valuable clues into the underlying mechanisms of cuproptosis in HCC and may help predict patients’ survival more accurately.

## Methods

### Patients and datasets

The fragments per kilobase of transcript per million mapped reads (FPKM)-standardized RNA-seq data of 424 samples, including 50 normal hepatic tissues and 374 tumor samples, and the corresponding clinical and prognostic data were downloaded from the TCGA website (https://portal.gdc.cancer.gov/projects/TCGA-LIHC). Patients with unknown clinical information and an overall survival time of less than 30 days were excluded. Then, Ensembl IDs were processed and converted to official gene symbol, including lncRNAs, protein-coding genes, miRNAs, etc.

### Identification of cuproptosis-related lncRNAs

To identify the CRLs, a total of 13 cuproptosis-related genes (CRGs) were summarized from recently published literature (10) (**Supplementary Table S1**). According to previous documents, Pearson analysis was considered an accepted method to investigate the correlation between coding genes and lncRNAs in the RNA-seq data of TCGA HCC samples (18, 19). Before obtaining enough cuproptosis-related lncRNAs, we have set various R values based on the published documents (18, 20, 21) and chosen R > 0.4 and P < 0.001 as the best cutoff value eventually. Then, the associations between CRLs and CRGs were initially filtered.

### Differential expression analysis of lncRNAs

The expression levels of CRLs between HCC and normal hepatic tissues were examined using the Wilcoxon test. A false discovery rate (FDR) < 0.01 and |Fold change (FC)| > 2 were set as screening criteria to obtain differentially expressed lncRNAs (DELs).

### The construction of the co-expression network

The co-expression network between CRLs and CRGs was constructed by Cytoscape software (version 3.7.2). Then, the ggalluvial R software package was used to plot a Sankey diagram in order to demonstrate the degree of correlation between CRLs (risk/protect) and their corresponding CRGs.

### The construction of cuproptosis-related prognostic signature

By using the ‘survival’ R package and defining *p* < 0.05 as screening criteria, the intersecting lncRNAs of CRLs and DELs were subsequent to univariate cox analysis for obtaining prognostic CRLs in HCC patients. least absolute shrinkage and selection operator (LASSO) Cox regression analysis was applied to construct CRLs predictive signature (22, 23). Initially, 18 prognostic lncRNAs were screened out on the basis of the optimal penalty parameter λ determined by tenfold cross-validation following the minimum criteria. Afterwards, multivariate cox regression analysis was conducted for the establishment of a five-lncRNA predictive model. The computational formula used for cuproptosis-related prognostic risk score was as follows:

Risk score = Coef_i_ lncRNA1 × lncRNA1 expression + Coef_i_ lncRNA2 × lncRNA2 expression + · ···· +Coef_i_ lncRNAn × lncRNAn expression. Coef_i_ represents the coefficient value of the corresponding lncRNA. Based on the median value of the risk score, patients were divided into low-risk and high-risk groups. The R package ‘survminer’ was used to generate the Kaplan–Meier curve with a log-rank test to compare the prognostic significance of cuproptosis-related five-lncRNA risk model. To assess the predictive ability of the lncRNA-based prognostic risk signature, the R package “timeROC” was used to examine the receiver operating characteristic (ROC) of 1/3/5-year survival (24). Moreover, univariate and multivariate Cox regression methods were performed to evaluate the prognostic prediction power of this risk score model.

### Construction of nomogram

A nomogram for predicting the 1-, 3-, and 5-year survival of HCC patients was developed using the risk model in conjunction with clinicopathological parameters such as age, gender, grade, stage, metastasis (M), positive lymph node (N) and vascular invasion. To determine if the anticipated survival rate was congruent with the observed survival rate, we employed a calibration curve.

### Function enrichment analysis

For the purpose of investigating the molecular mechanism and biological process involved in the cuproptosis-related lncRNA signature, GSEA was performed to discover which pathway genes were mainly enriched between the high/low risk groups using the h.all.v7.4 symbols.gmt [Hallmarks], and the Kyoto Encyclopedia of Genes and Genomes (KEGG) dataset c2.cp.kegg.v7.4.symbols.gmt from the molecular signature dataset (https://www.gsea-msigdb.org/gsea/msigdb) as references (25). The criteria for statistical significance were nominal *p* < 0.05 and FDR<0.25.

### Immune infiltrate analysis

CIBERSORT is an analytical tool that uses preprocessed gene expression profiles to depict the cell composition of complex tissues (26). 22 different kinds of TIICs were examined using CIBERSORT. To compare the fraction of tumor-infiltrating immune cells, the Wilcox test was performed. Spearman’s correlation test was used to examine the link between the signature’s risk score and immune cells. The relationship between the signature’s risk score and the expression of immune checkpoint genes was determined by Pearson’s test.

### The role of the risk signature in predicting drug sensitivity

Predictions of drugs’ IC50 values were made using the ‘pRRophetic’ R program (27), which calculates the efficiency of Elesclomol in provoking cuproptosis.

### HCC cell lines, human samples and reagents

Researcher Tong Ji, an assistant of the principal investigator at Cang laboratory at Zhejiang University, provided HCC cells, including HA22T and Huh7. Normal liver cell, MIHA, was kindly provided by Dr J R Chowdhury (Albert Einstein College of Medicine, New York). All cell lines were cultured in DMEM medium (Gibco, USA) containing 10% fetal bovine serum (FBS, Gibco) at 37°C in humidified air with 5% CO2. A total of 36 HCC samples and adjacent normal tissues were collected from HCC patients who underwent surgical resection at the Sir Run Run Shaw Hospital (SRRSH). All patients involved provided written informed consent. This research was approved by the Institutional Review Board of the SRRSH, School of Medicine, Zhejiang University, Hangzhou, China. Elesclomol (S1052), Z-VAD-FMK (ZVF, S7023), ferrostatin-1 (Fer-1, S7243), necrostatin-1 (Nec-1, S8037), and N-acetyl cysteine (NAC, S5804) were purchased from Selleck. Tetrathiomolybdate (TTM, 323446) was purchased from Sigma.

### RNA interference

The Ribo™ Smart Silencer, composed of three anti-sense oligonucleotides (ASO) and three small interference RNAs (siRNAs) was designed and synthesized in Ribobio (China), which could effectively knock down both nuclear and cytoplasmic lncRNAs. Cells were transfected with 100 nM of smart silencer for each well using the Lipofectamine™ 3000 transfection reagent (L3000015; Thermo Fisher, USA). After 48 hours of transfection, cells were collected and processed for RT-qPCR and other experiments. The sequences of the lncRNA smart silencer were listed in **Table 1**.

**Table 1:** Sequences of lncRNA smart silencers.

Oligonucleotides	Sequence (5’-3’)
siRNA targeted sequence: NRAV	TCCGAGCAACACCTAAACAA
ASO targeted sequence: NRAV	CTAGGTCTGAATCCGGAAGC
siRNA targeted sequence: NRAV	ACAACTCAGGGAAAGAAAAC
ASO targeted sequence: NRAV	GGATGGATAGTTCAGAGTA
siRNA targeted sequence: NRAV	GATCTTCCTTGGACAGAAT
ASO targeted sequence: NRAV	GAGCAACAATTACAGATCA
siRNA targeted sequence: WARS2-AS1	GGAGAGAAATAAATAGAGG
ASO targeted sequence: WARS2-AS1	GTCTAAGAAGGAAATGTGA
siRNA targeted sequence: WARS2-AS1	GAGCCGTCTTGTTTGGAAT
ASO targeted sequence: WARS2-AS1	CTTATGAAGTGCCCGGATGA
siRNA targeted sequence: WARS2-AS1	GCCGTCTTGTTTGGAATGAC
ASO targeted sequence: WARS2-AS1	TTGGAAGATGGAAGAGGACC
siRNA targeted sequence: MKLN1-AS	GCCACACTTTGATCCTAAA
ASO targeted sequence: MKLN1-AS	CCCATCTAACCTGGAATGA
siRNA targeted sequence: MKLN1-AS	CACCTTCATTCAAGAGGAA
ASO targeted sequence: MKLN1-AS	TGGCCTGGTCCCTTGTCTAC
siRNA targeted sequence: MKLN1-AS	ACAAGCAGAGCCACTGCAGT
ASO targeted sequence: MKLN1-AS	GCCTGGACAGTGTCATCATC

ASO, antisense oligonucleotides.

### RNA extraction and quantitative real-time PCR

RNA extraction was performed using the RNA-Quick Purification Kit (ES Science) following the manufacturer’s instructions. Reverse transcription was conducted according to the Eco M-MLV RT Premix kit (AG11706, Accurate Biology). Target gene expression was normalized to the endogenous control gene glyceraldehyde 3-phosphate dehydrogenase (GAPDH). RT-qPCR was conducted on the QuantStudio 1 (applied biosystems, Thermo Fisher Scientific, USA) machine utilizing SYBR Green Premix Pro Tag HS qPCR kit (AG11701, Accurate Biology). Expression levels of lncRNAs were calculated with 2^−ΔΔCT^ method. **Table 2** showed the primer sequences used for RT-qPCR.

**Table 2:** Primers used in this study.

Primer name	Sequence (5’-3’)
GAPDH-F	TTGGTATCGTGGAAGGACTCA
GAPDH-R	TGTCATCATATTTGGCAGGTT
NRAV-F	TGAGGGATTCCTTACGGGGT
NRAV-R	AGGGTGGTCACAGGACTCAT
MED8-AS1-F	CTGCCTGCATCTAGACTGTTCT
MED8-AS1-R	TTGACACGCAGGCCAGTTTT
WARS2-AS1-F	CAGTTCTTGCCGAGCAGTCG
WARS2-AS1-R	CTTCTAAATGCCACAGCACCTG
MKLN1-AS-F	AGAGGTTGGACTCCTGAAAGC
MKLN1-AS-R	CCAGACCCTCGTATTACGTCC
FOXD2-AS1-F	TATGTGGTAGGGGACTCGCT
FOXD2-AS1-R	GGTTTCAAGTGGCGCTGTTT

### Cell viability

A total of 3000-4000 HCC cells per well were planted into a 96-well plate and allowed to attach for 16-24h. Then HCC cells were exposed to increasing doses of elesclomol for 24h. Then, 100 μL of fresh medium containing 10% CCK8 solution (MA0218, Meilunbio, China) was added, and the 450-nm absorbance was detected following incubation for 1.5h at 37°C using a spectrophotometer (Multiskan Spectrum 1500, Thermo, USA). Where specified, indicated concentrations of Cucl_2_ were added to the media. As for the chemical rescue assay, cell death inhibitors were added after plating for 6h, then cuproptosis inducer elesclomol was added into plates and incubated for 48h.

### Statistical analysis

RStudio and its associated packages were used to conduct all statistical analyses. The ‘ggplot2’ package was utilized to visualize the graphs. The Wilcox test was used to compare lncRNA expression between HCC and para-noncancerous tissues of TCGA. The chi-squared test was used to examine differences in the proportions of clinical features. A paired t-test was used to analyze the data between HCC and adjacent normal tissues in house. Variances among multiple groups were analyzed by one-way ANOVA. Statistical significance was defined as a *p*-value<0.05.

## Results

### Identification of cuproptosis-related differentially expressed lncRNAs in HCC

**Figure 1:**
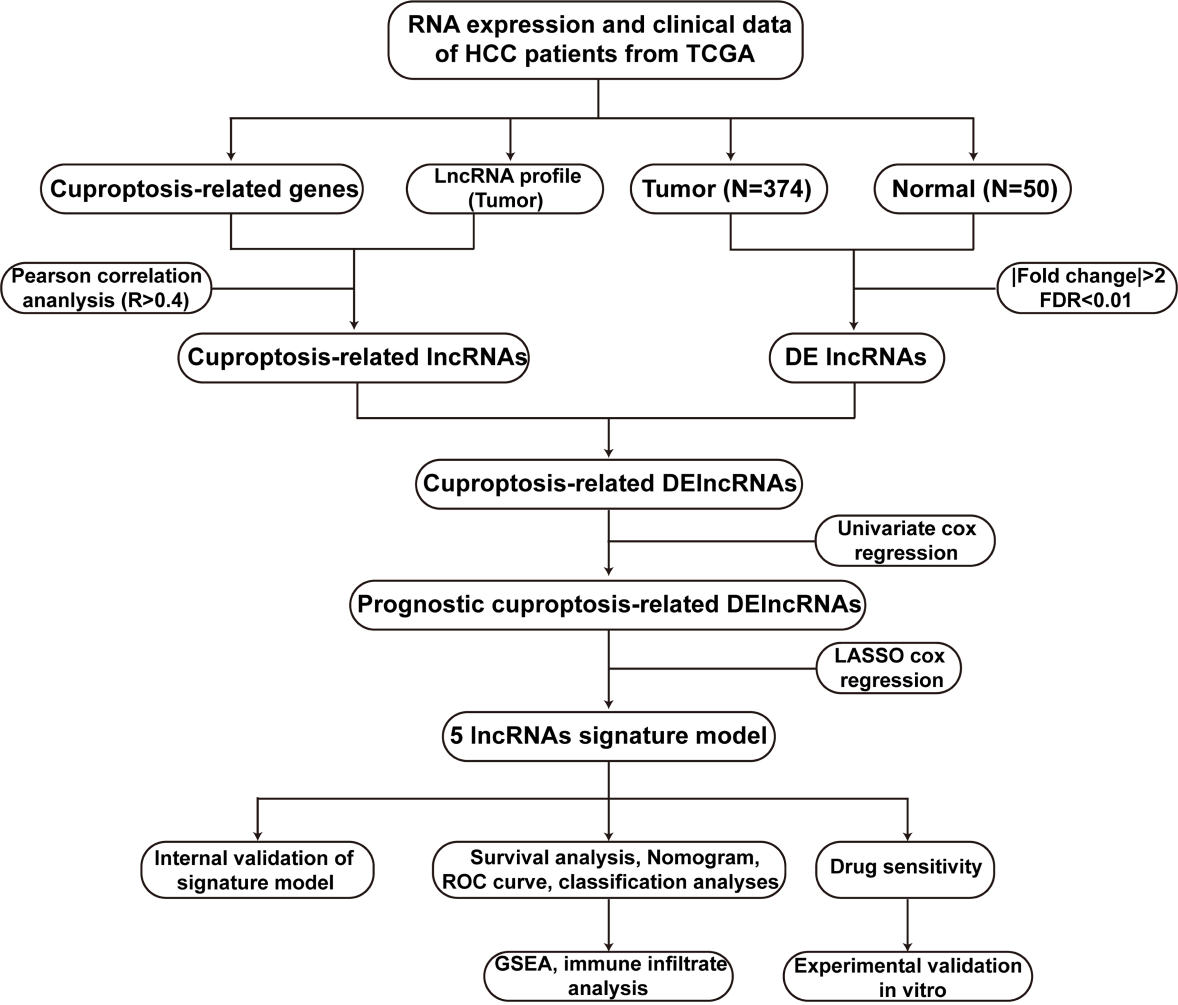
The flowchart of this work. HCC, hepatocellular carcinoma; TCGA, The Cancer Genome Atlas; DElncRNAs, differentially expressed lncRNAs; lncRNAs, long noncoding RNAs; ROC, receiver operating characteristic; GSEA, gene enrichment analysis.

**Figure 2:**
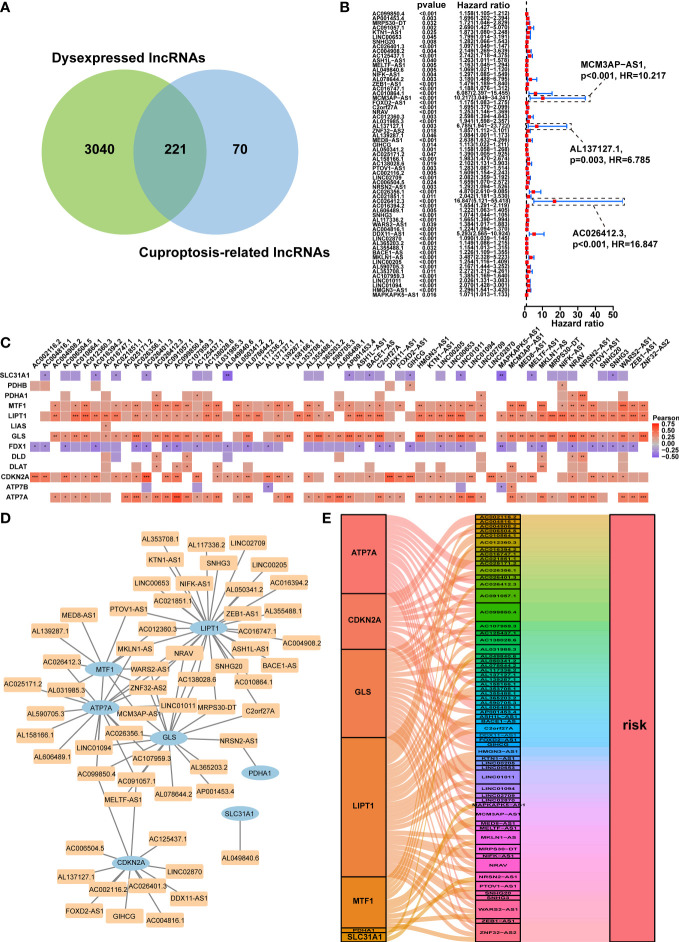
Prognostic analysis of differentially expressed cuproptosis-related lncRNAs and the construction of a coexpression network. **(A)** Venn diagram identifying the lncRNAs shared by differentially expressed lncRNAs and cuproptosis-related lncRNAs. **(B)** Forest plots displaying the outcomes of the univariate cox regression analysis of about 61 prognostic differentially expressed CRLs. **(C)** The correlation between 61 prognostic CRLs and 13 CRGs in the TCGA-HCC cohort. Each unit’s color indicated the degree of correlation. Red implied the positive relationship, blue was on the contrary. **(D)** Coexpression network of 61 prognostic differentially expressed CRLs and CRGs based on the Pearson’s R>0.4 and P<0.001. **(E)** The Sankey diagram illustrated the link between the 61 prognostic differentially expressed CRLs and CRGs on the basis of Pearson’s R>0.4 and P<0.001. lncRNAs, long noncoding RNAs; CRLs, cuproptosis-associated lncRNAs; CRGs, cuproptosis-related genes; HCC, hepatocellular carcinoma. *p < 0.05, **p < 0.01, and ***p < 0.001.

**Figure 1** illustrated the research flow diagram for our investigation. The FPKM-standardized RNA-seq data of 424 samples were downloaded from TCGA. Then, a total of 14086 lncRNAs and 19604 mRNAs were separated, respectively. According a recently published literature (10), authors have proposed a novel cell death named cuproptosis in various diseases context, which was not reported previously. We obtained 13 CRGs from the study and listed their detailed information in **Supplementary Table 1**. To evaluated CRLs, the correlation between CRGs and lncRNAs was performed *via* Pearson analysis on the basis of R>0.4 and *p* value<0.001. There were a total of 291 CRLs defined in total. Meanwhile, 3261 lncRNAs with differential expression were discovered in HCC samples compared to the normal tissues (log2|FC| > 1, FDR < 0.01) (**Supplementary Table 2**). **Supplementary Figure 1** depicted a volcanic map of DELs. Moreover, combined with CRLs and DELs, we obtained a total of 221 cuprotosis-related differentially expressed lncRNAs (CRDELs) (**Figure 2A**).

### Identification of prognostic cuproptosis-related differentially expressed lncRNAs

The predictive ability of the CRDELs was examined by univariate Cox regression analysis utilizing the overall survival (OS) data of HCC patients in the TCGA database. This led to the identification of 61 prognostic CRDELs (**Figure 2B** and **Supplementary Table S3**). All of these lncRNAs were regarded as “risk” genes. As shown in **Figure 2C**, the correlation between the prognostic CRDELs and CRGs was plotted. In addition, the detailed correlation rate and *p* value between each prognostic CRDEL and CRG were listed in **Supplementary Table 4**.

Furthermore, a lncRNA-gene coexpression network was created in order to further assess the relationship between these 61 prognostic CRDELs and the representative CRGs based on the Pearson’s R>0.4 and *p*<0.001 in great detail (**Figure 2D**). Among these lncRNAs, two lncRNAs (WARS2-AS1 and ZNF32-AS2) were co-expressed with 4 CRGs including LIPT1, MTF1, GLS, ATP7A, while AC099850.4 was co-expressed with MTF1, GLS, CDKN2A, ATP7A. Among CRGs, lipoyltransferase 1 (LIPT1), exhibited positive coexpression with 29 prognostic CRDELs significantly. Additionally, ATPase Cu transporting alpha (ATP7A) was linked to 17 prognostic CRDELs. **Supplementary Table 5** provided further information on the coexpression network in detail. Following that, the intrinsic link between prognostic CRDELs and CRGs was later revealed *via* visualizing their prognostic function. CRDELs, CRGs and their roles in HCC were shown in a Sankey diagram that we created (**Figure 2E**).

### Construction of the prognostic signature based on cuproptosis-associated five-lncRNAs

**Figure 3:**
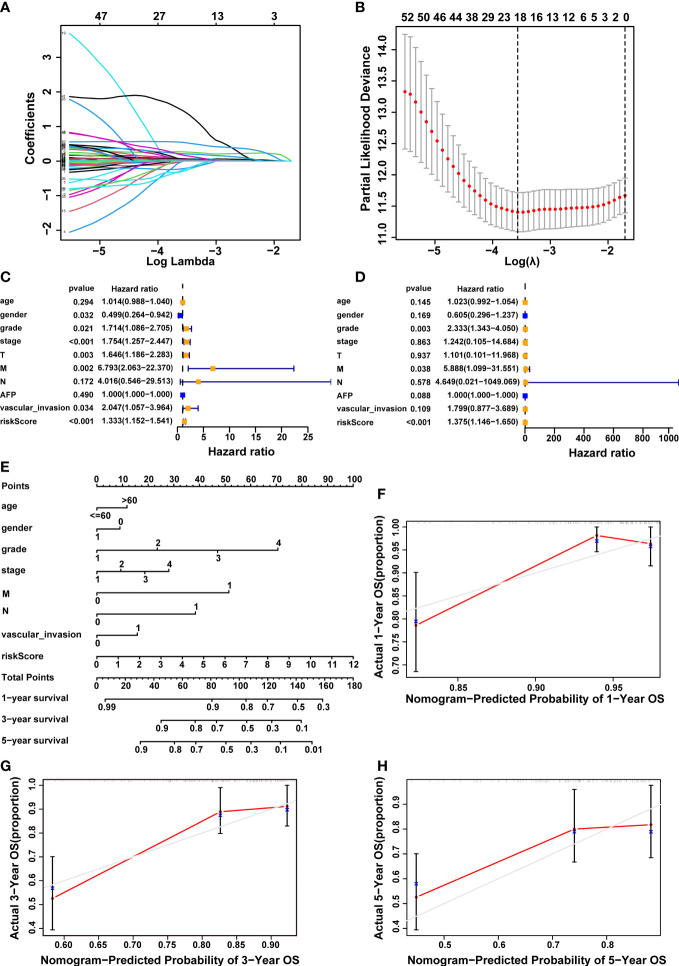
Construction of a 5-cuproptosis-related-lncRNA signature and evaluation of its predictive value. **(A, B)** Cvfit and lambda curves demonstrating LASSO regression generated using a 10-fold cross-validation. **(C, D)** Results of the univariate and multivariate independent prognostic analysis in addressing the 5-cuproptosis-related lncRNA signature’s overall survival. **(E)** The nomogram model of age, gender, grade, stage, M, N, vascular invasion and risk score was used to forecast the 1-year, 3-year, and 5-year overall survival rate of HCC patient. **(F-H)** The calibration curves evaluated the congruence between the observed OS rates and the expected survival rates at 1, 3, and 5 years. The dashed grey diagonal line was the optimal nomogram. lncRNAs, long noncoding RNAs; LASSO, Least absolute shrinkage and selection operator; OS, overall survival; HCC, hepatocellular carcinoma; M, metastasis, N, lymph node.

To build a prognostic model utilizing the expression profiles of 61 CRDELs described above, LASSO Cox regression analysis was used. **Figures 3A, B** showed the cvfit and lambda curves, respectively, based on multivariate cox regression analysis, the five-lncRNA expression risk score for each sample was calculated as follows: 0.475 × FOXD2-AS1 expression + 0.631× NRAV expression + 0.903 × MED8-AS1 expression + -1.24 × WARS2-AS1 expression + 1.362 × MKLN1-AS expression. Univariate and multivariate Cox regression analyses were performed among the clinical variables to examine whether the predictive signature was an independent prognostic factor for HCC patients. Univariate cox regression analysis found that gender, grade, stage, T stage, M stage, vascular invasion, and risk score were all significant predictors of OS in patients with HCC (**Figure 3C**). The results of a multivariate cox regression analysis revealed that grade, M stage and risk score were independent determinants of OS in HCC patients (**Figure 3D**).

In order to better forecast the prognosis of HCC patients, we developed a nomogram that included different clinicopathological characteristics as well as the risk score. This nomogram was capable of predicting the prognosis of HCC patients at 1, 3, and 5 years (**Figure 3E**). Furthermore, the calibration curves demonstrated a high degree of congruence between the observed and anticipated survival rates at 1, 3, and 5 years (**Figures 3F–H**).

**Figure 4:**
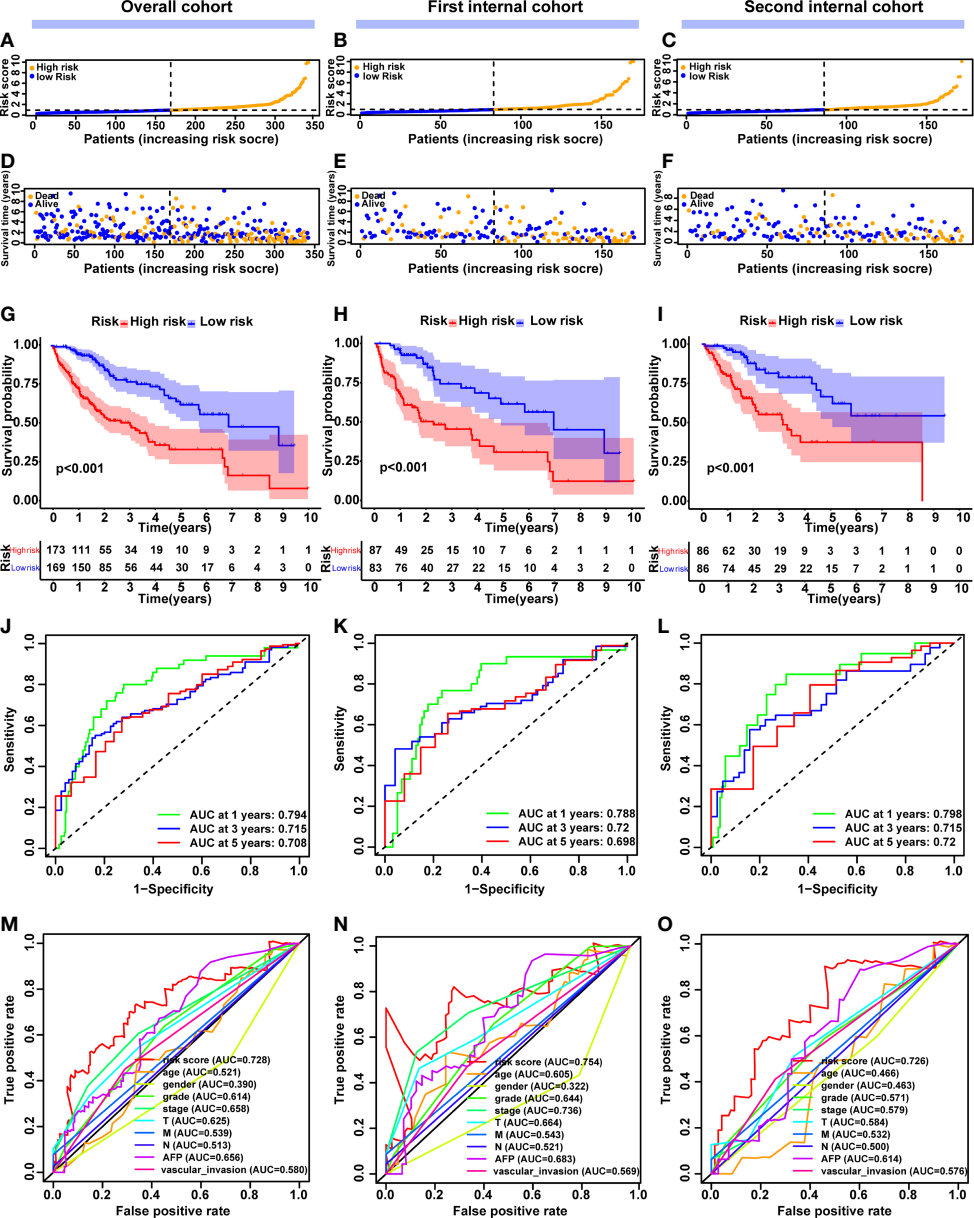
Construction and validation of the cuproptosis-related lncRNA signature model in the overall, first internal and second internal cohorts. **(A-C)** The distribution and median value of the risk scores in the overall, first internal and second internal cohorts. **(D-F)** The distribution of overall survival status, survival time and risk score in the overall, first internal and second internal cohorts. **(G-I)** The Kaplan–Meier curves for survival status and survival time in the overall, first internal and second internal cohorts. **(J-L)** AUC of time-dependent ROC curves demonstrated the ability of the signature of prognostic cuproptosis-related lncRNAs to predict 1-, 2-, and 3-year OS in the overall, first internal and second internal cohorts. **(M-O)** AUC of ROC curves comparing the prognostic accuracy of the lncRNA signature model and other prognostic parameters in the overall, first internal and second internal cohorts. lncRNAs, long noncoding RNAs; ROC, receiver operating characteristic; AUC, area under the curve; OS, overall survival.

Following that, we focused on the prognostic value of this 5-CRL model to evaluate how well it performed. The median cut-off value was used to divide the patients into high-risk and low-risk groups (**Figure 4A**). As the risk score increased, an increasing number of HCC patients died (**Figure 4D**). The Kaplan-Meier method was used to compare the OS time of the high- and low-risk groups. In comparison to the low-risk group, the high-risk group’s OS time was much shorter (**Figure 4G**). Intriguingly, good predictive performance was shown by the area under the curve (AUC) of survival at 1, 3, and 5 years, which were 0.794, 0.715, and 0.708 respectively (**Figure 4J**). For further validation, an ROC curve was built to demonstrate the signature’s superior predictive accuracy in comparison with other clinicopathological parameters (**Figure 4M**). To assess the prognostic signature for OS’s applicability throughout the overall TCGA dataset, 342 HCC patients were randomly separated into two cohorts *via* 1:1 ratio. Consistent with the results observed in the entire dataset, there was a reasonable distribution of the samples from the two risk categories in the first (**Figures 4B, E, H, K, N**) and second internal cohort (**Figures 4C, F, I, L, O**). Taking all of these studies together, it was shown that this unique cuproptosis-associated five-lncRNA signature could be a reliable independent predictive factor for patients with HCC.

### Relationship between the 5-CRL signature and the clinicopathological characteristics in HCC patients

**Figure 5:**
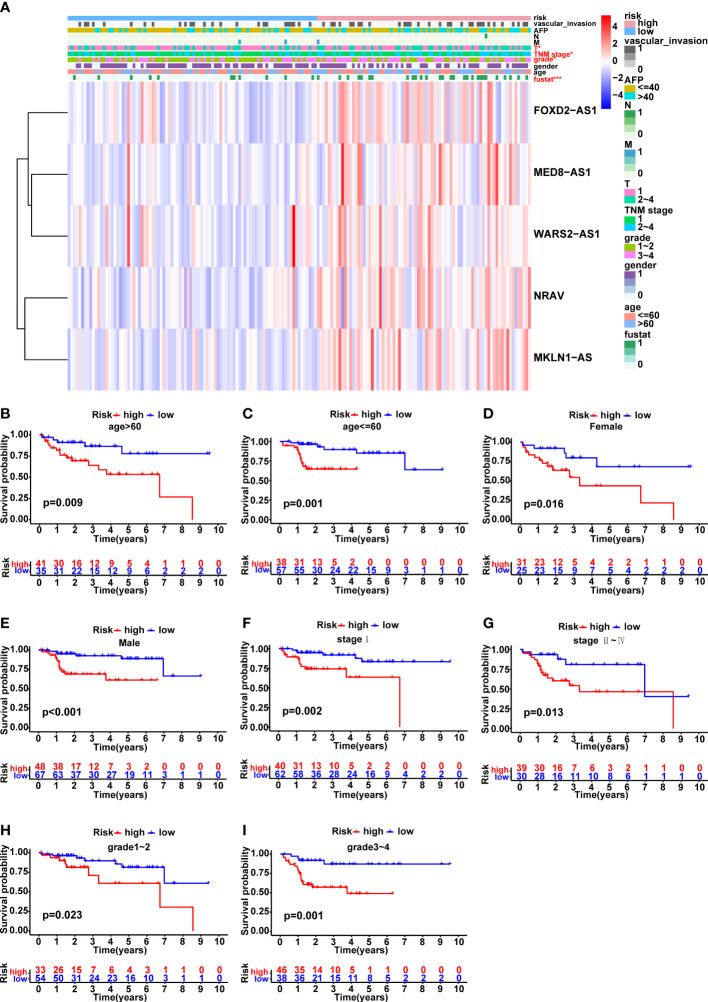
Correlation analysis between the prognostic signature and different clinicopathological characteristics in the TCGA cohort. **(A)** The heatmap illustrating the distribution of ten distinct clinicopathological features, together with the risk score for each patient based on the predictive signature. Clinicopathological features in red indicated that there was an obvious difference distributed in the high- and low-risk group. **(B-I)** Kaplan-Meier survival curves for high-risk and low-risk patient groups based on age, gender, TNM stage and grade classification. *p < 0.05, and ***p < 0.001.

The variations in clinicopathological parameters between the two risk groups were compared (**Figure 5A**). Interestingly, there were significant differences in fustat (*p*<0.001), T stage (*p*<0.05), TNM stage (*p*<0.05) and grade (*p*<0.05) between the high- and low-risk groups. In addition, HCC patients were classified into groups according on age, gender, AFP, grade, M stage, T stage, N stage, TNM stage, and vascular invasion in order to explore the correlation between the predictive signature and the prognosis of HCC patients. It was clear that for each group, the OS time of patients at high risk was considerably shorter than that of individuals in the low-risk group (**Figures 5B–I** and **Supplementary Figures 2A–H**). Due to the small number of patients in N1 and M1 groups, the survival curves were not presented here. Altogether, these findings implied that the predictive signature may accurately predict the outcome of HCC patients without taking clinicopathological characteristics into account.

### Correlation between the predictive signature and cuproptosis

**Figure 6:**
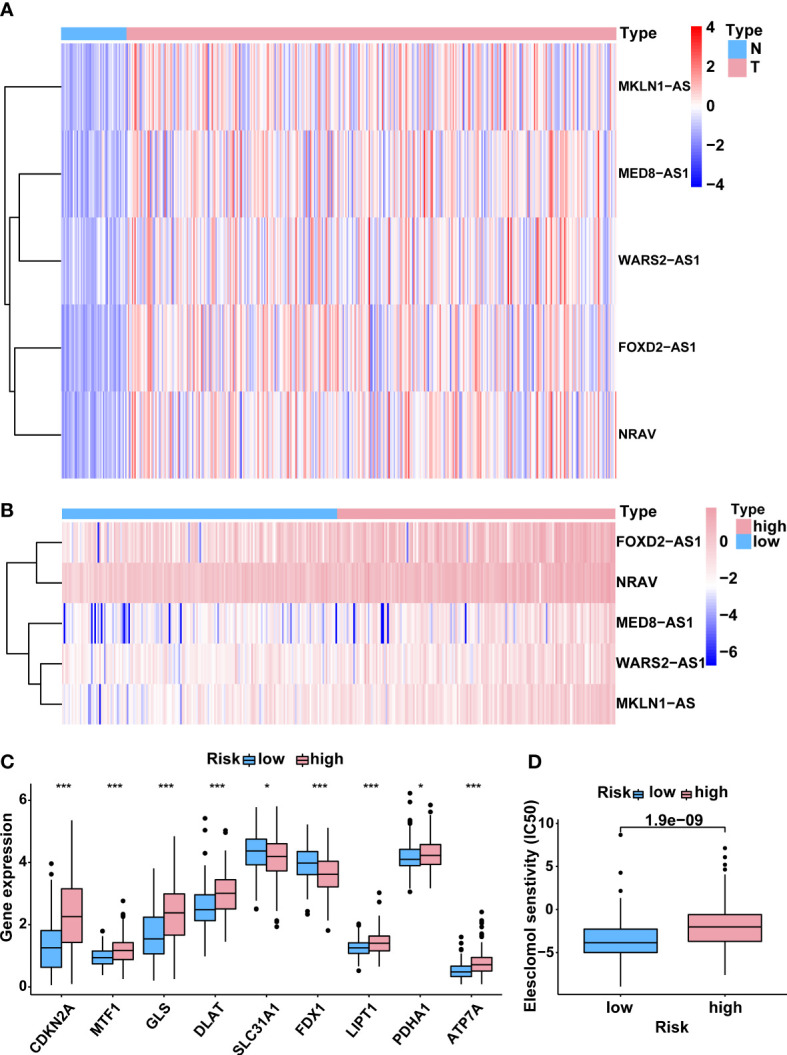
Correlation between the predictive signature and cuproptosis. **(A)** The expression levels of 5 lncRNAs associated with cuproptosis in HCC and normal tissues. **(B)** The expression levels of 5 cuproptosis-related lncRNAs in groups with low and high risk. **(C)** The differential expression levels of CRGs between high- and low-risk groups. **(D)** Comparison of senstivity of cuproptosis inducer elesclomol between high- and low-risk groups. lncRNAs, long noncoding RNAs; HCC, hepatocellular carcinoma; CRGs, cuproptosis-related genes. *p < 0.05, and ***p < 0.001.

Based on the risk model, a heatmap of the expression levels of five cuproptosis-related lncRNAs signature was depicted (**Figure 6A**). Meanwhile, the heat map of potential lncRNAs signature profiles revealed that FOXD2-AS1, NRAV, MED8-AS1, WARS2-AS1 and MKLN1-AS were all significantly increased in the high-risk group (**Figure 6B**). In order to investigate the relationship between the five-lncRNA model and cuproptosis, the differential expression of all 13 CRGs was compared between the two groups. Results indicated that most CRGs were obviously dysexpressed with significant *p* value (**Figure 6C**). Furthermore, Cu ionophore like elesclomol was small molecule that bound Cu and transported it into cells, making it valuable for investigating Cu toxicity (8, 10). To better understand the sensitivity of differences between the two risk groups in terms of the cuproptosis inducer elesclomol, the estimated IC50 value of elesclomol was lower in the low-risk group, which was beneficial for developing tailored treatment strategies for individuals in the low-risk groups (**Figure 6D**). Collectively, these results implied that this five-lncRNA signature might exert a vital role during the process of cuproptosis, which provided a novel clue for us to explore this unique cell death in HCC.

### GSEA enrichment analysis

**Figure 7:**
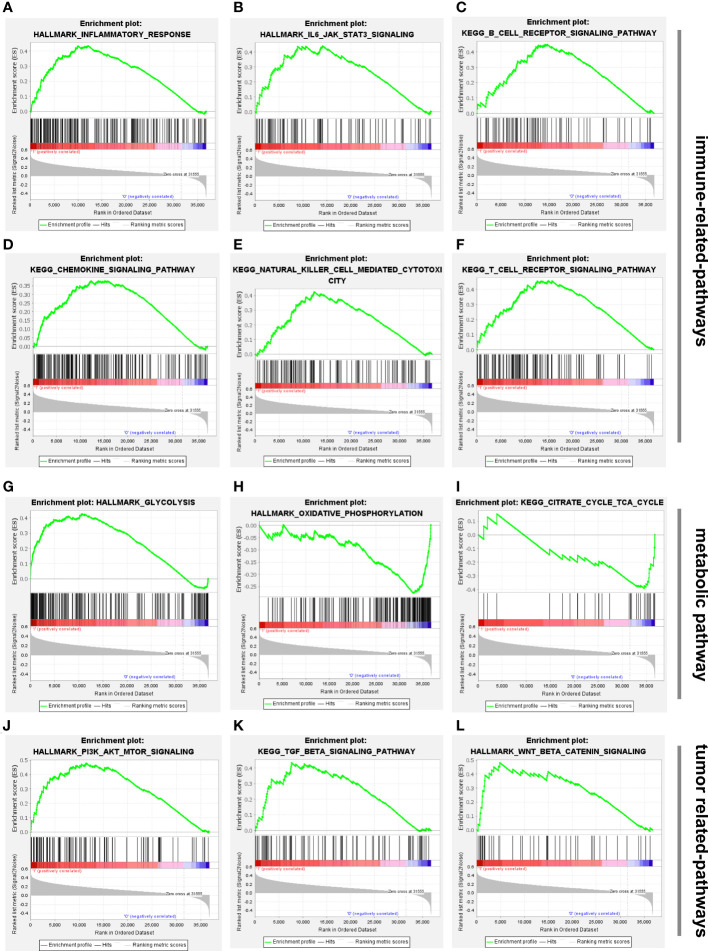
Biological functional and pathway enrichment analysis of high-risk group and low-risk group based on the cuproptosis-associated lncRNA signature *via* GSEA analysis. **(A-F)** GSEA showing significant enrichment of immune-related pathways in the high-risk HCC patients, including inflammatory response, IL6/JAK/STAT3 signaling, B cell receptor signaling pathway, chemokine signaling pathway, natural killer cell mediated-cytotoxicity and T cell receptor signaling pathway. **(G-I)** Glycolysis was mainly enriched in the high-risk group while oxidative phosphorylation and citrate cycle TCA cycle were related to low-risk group. **(J-L)** GSEA showing significant enrichment of tumor-related pathways in the high-risk HCC patients, including PI3K/AKT/mTOR, TGF-β signaling and wnt/β/cantenin pathways. lncRNAs, long noncoding RNAs; HCC, hepatocellular carcinoma; GSEA, gene enrichment analysis.

To better understand the biological processes and pathways linked with the two risk groups identified using the 5-CRL signature, GSEA was conducted. Results of GSEA analysis indicated that several immune-associated signaling pathways were enriched in the high-risk group, including inflammatory response, IL6/JAK/STAT3 signaling, B cell receptor signaling pathway, chemokine signaling pathway, natural killer cell mediated-cytotoxicity and T cell receptor signaling pathway (**Figures 7A–F**). More surprisingly, glycolysis was mainly correlated with high-risk groups while pathways related to the low-risk group were mainly involved in the regulation of oxidative phosphorylation and the citrate cycle TCA cycle (**Figures 7G–I**). Since oxidative phosphorylation and the TCA cycle were the main biological processes associated with cuproptosis (10), it is convincible to us that the five lncRNAs might play a critical role in the regulation of this unique cell death. Apart from those, numerous cancer malignant phenotypes-associated pathways, including PI3K/AKT/mTOR, TGF-β signaling and wnt/β/cantenin pathways, were more prevalent in the high-risk group (**Figures 7J–L**). The details of the GSEA results are listed in **Supplementary Table 6**.

### Immunity analysis of the risk score

**Figure 8:**
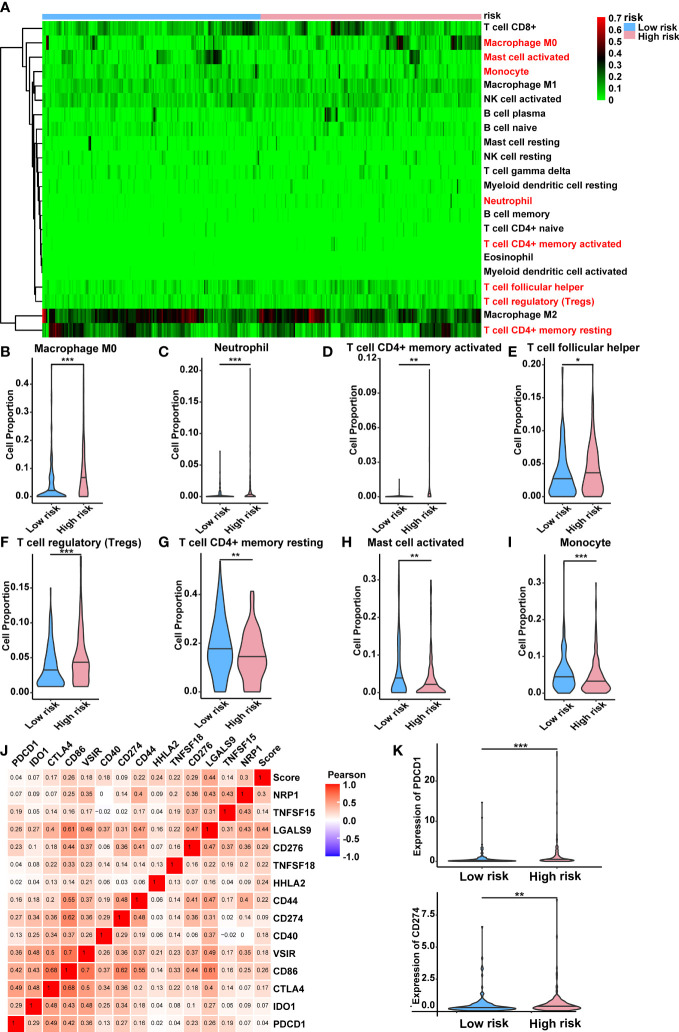
Relationship between the lncRNA-based signature and immune responses in HCC. **(A)** Relative proportion of 22 different immune cells based on CIBERSORT in the low-risk group and the high-risk group. Immune cells in red indicated that there was a significant difference within the two groups. **(B-I)** The proportion of M0 macrophages, neutrophil, activated memory CD4+ T cells, follicular helper T cells, regulatory T cells, activated mast cell, monocyte and resting memory CD4+ T cells in the low-risk group and the high-risk group. **(J)** Heat map showing the relations between risk score and various immune checkpoints *via* Pearson test. **(K)** PDCD1 and CD274 were significantly upregulated in the high-risk group. lncRNAs, long noncoding RNAs; HCC, hepatocellular carcinoma. *p < 0.05, **p < 0.01, and ***p < 0.001.

For the purpose of determining whether this five-lncRNA signature was associated with tumor immunity, this signature was then examined in relation to the 22 types of TIICs in HCC that were identified using the CIBERSORT algorithm (**Supplementary Figure 3**). As illustrated in **Figure 8A**, the CIBERSORT-based heat map of immune responses revealed that M0 macrophages, activated mast cell, monocyte, neutrophil and T cell functions, including resting/activated memory CD4+ T cells, follicular helper T cells, and regulatory T cells, were all significantly different between the high-risk and low-risk groups. A considerably greater proportions of M0 macrophages, neutrophil, activated memory CD4+ T cells, follicular helper T cells, and regulatory T cells was found in the high-risk group while the low-risk group displayed a lower percentage of activated mast cell, monocyte and resting memory CD4+ T cells and (**Figures 8B–I**). These findings suggested that the infiltration of these immune cell subtypes into the tumor microenvironment might have a significant impact on the prognosis of HCC patients.

Moreover, the clinical significance of immune therapy strategies in HCC prompted us to explore the link between the risk score and a number of immune checkpoints (28). This was shown by the heat map in **Figure 8J**, which indicated a positive relationship between the risk score and various immune checkpoints. Among them, numerous immunotherapy targets that have been shown to be beneficial in clinical, such as PDCD1 (PD-1), CD274 (PD-L1), were expressed at a high level in the high-risk group (**Figure 8K**). Additionally, we have explored the correlation between differentially expressed CRGs within two risk groups and immune checkpoints. Results indicated that most CRGs were significantly co-expressed with immune checkpoints, which established a link between cuproptosis and immune response directly (**Supplementary Figure 4**). The detailed correlation rate and *p* value between each CRG and immunity gene were listed in **Supplementary Table 7**.

### Validation of five cuproptosis-related lncRNAs expression in HCC cell and tissues

**Figure 9:**
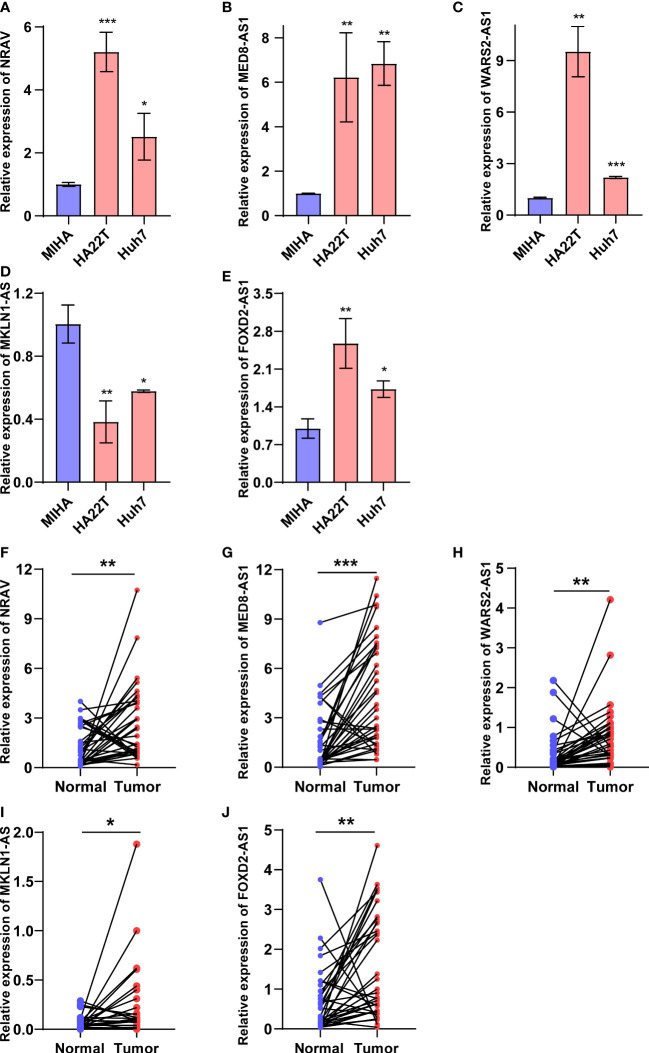
Validation of five cuproptosis-related lncRNAs expressions in HCC cell and tissues. **(A-E)** FOXD2-AS1, NRAV, MED8-AS1, WARS2-AS1 and MKLN1-AS expression in normal liver cells and HCC cells. **(F-J)** FOXD2-AS1, NRAV, MED8-AS1, WARS2-AS1 and MKLN1-AS expression in HCC tissues and adjacent normal tissues. *p < 0.05, **p < 0.01, and ***p < 0.001.

The five cuproptosis-related lncRNAs expression in HCC cells and tissue were detected through RT-qPCR assay. As shown in the **Figures 9A–E**, compared to the normal liver cell MIHA, the expression levels of FOXD2-AS1, NRAV, MED8-AS1, and WARS2-AS1 were significantly upregulated in HCC cells except MKLN1-AS. Furthermore, we also conducted RT-qPCR to validate their expressions in 36 pairs of HCC tissues and adjacent normal tissues. Results indicated that FOXD2-AS1, NRAV, MED8-AS1, WARS2-AS1 and MKLN1-AS exhibited higher expressions in HCC patients (**Figures 9F–J**). However, although the results of MKLN1-AS in tissues and cell lines differed, the RT-qPCR results in the tissue were more credible because the expression level in the cell line did not fully represent the RNA-seq data of TCGA. Overall, our experimental findings also validated the reliability of the predictive signature.

### Elesclomol-induced cuproptosis regulated by WARS2-AS1 and MKLN1-AS

**Figure 10:**
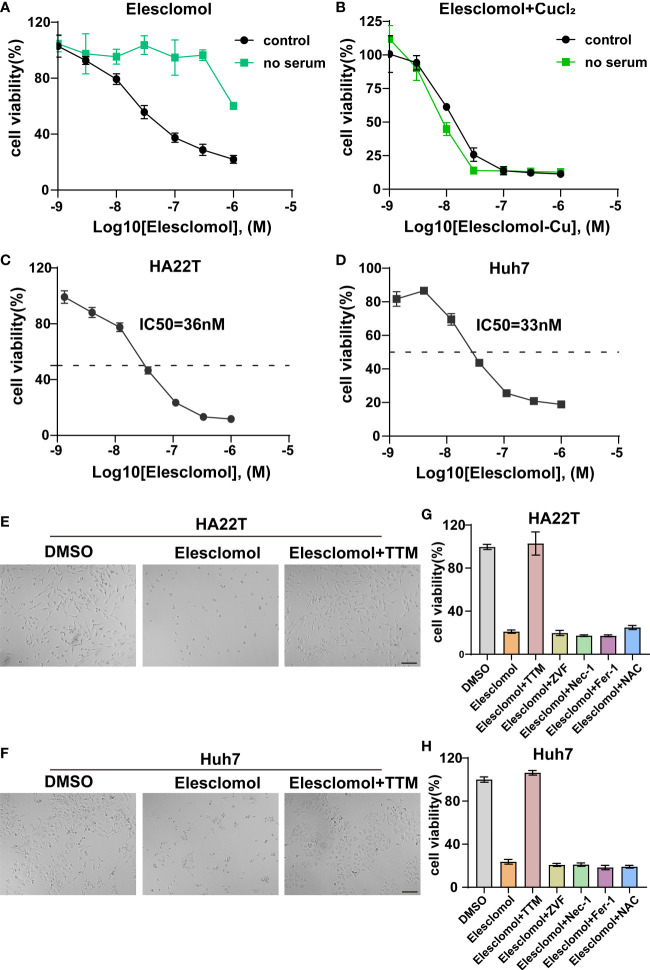
Validation of cuproptosis induced by elesclomol. **(A)** Cell viability of HCC cell when grown in the presence or absence of serum and treated with elesclomol. **(B)** Cell viability of HCC cell when grown in the presence or absence of serum and treated with either elesclomol or elesclomol in the presence of CuCl2. **(C, D)** HA22T and Huh7 cells were exposed to different doses of elesclomol for 24h and detected by CCK8 reagent. **(E, F)** Representative images of HCC cells treated with elesclomol (40nM) with or without TTM (5 μM) for 48h. Scale bars represent 200μm. **(G, H)** The rescue effect of cell death inhibitors on elesclomol treatment in HA22T and Huh7 was explored through CCK8 assay. Data was presented as mean+SD. ZVF, Z-VAD-FMK; Fer-1, ferrostatin-1; Nec-1, necrostatin-1; NAC, N-acetyl cysteine; TTM, Tetrathiomolybdate.

Accumulating evidence indicated that elesclomol was characterized as a Cu-binding small molecule and elesclomol-induced cell death was attributed to accumulation of intracellular Cu instead of the effect of elesclomol itself (29, 30). However, studies have reported that Cu ionophore elesclomol could provoke various cell death including apoptosis, ROS and ferroptosis (29–31). To date, the Cu-induced cytotoxicity in HCC has not been clearly elucidated. Firstly, we evaluated whether the elesclomol-induced HCC cell death was dependent on Cu itself. Since serum was the source of Cu, we therefore found that HCC was more resistant to elesclomol in the absence of serum (**Figure 10A**). On the contrary, the restoration of elesclomol sensitivity was due to the addition of Cu in the cell medium (**Figure 10B**). Next, the IC50 values of elesclomol in two HCC cell lines were assessed, which were 36nM and 33nM respectively (**Figures 10C, D**). Under the induction of elesclomol, the cell viability was almost rescued by Cu chelator TTM while other cell death pathway inhibitors, including ZVF, Nec-1, Fer-1 and NAC failed to reverse the cell death provoked by the Cu ionophore elesclomol (**Figures 10E, G**). Comparable results were also found in another HCC cell line Huh7 (**Figures 10F, H**). These results indicated that elesclomol-induced cell death was dependent on Cu accumulation and that this cell death was distinct from other traditional cell death mechanisms.

**Figure 11:**
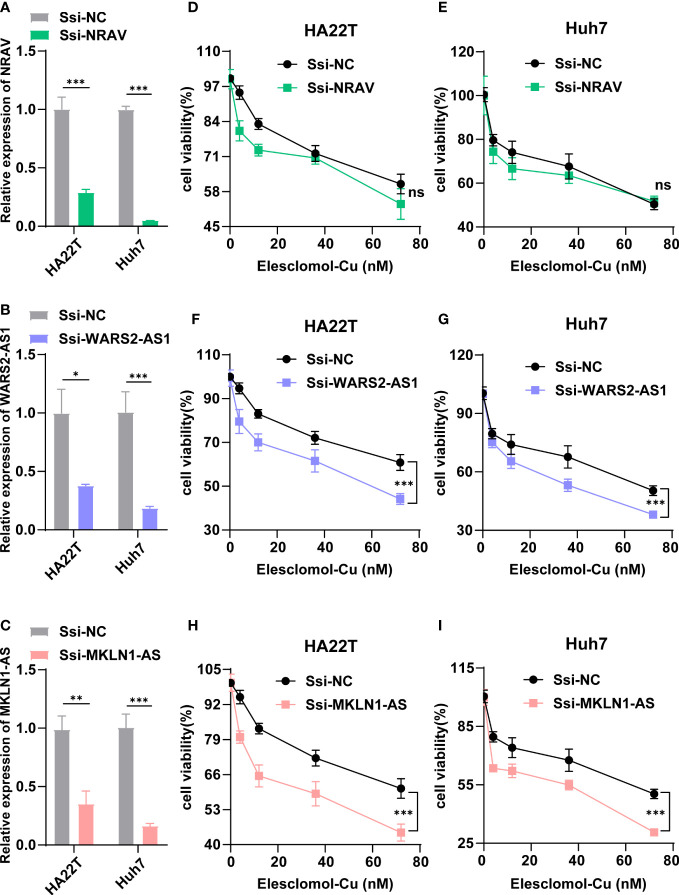
WARS2-AS1 and MKLN1-AS knockdown rendered cells susceptible for cuproptosis. **(A-C)** Relative NRAV, WARS2-AS1 and MKLN1-AS mRNA level in HCC cell with or without NRAV, WARS2-AS1 and MKLN1-AS knockdown (n=3). **(D-I)** HCC cells with or without NRAV, WARS2-AS1 and MKLN1-AS knockdown were exposed to different doses of elesclomol for 24h and detected by CCK8 reagent. SSi-NC, smart silencer negative control; *P < 0.05; **P < 0.01; ***P < 0.001; ns, no significant.

According to **Figure 2D**, three lncRNAs (NRAV, WARS2-AS1 and MKLN1-AS) in our signature were shown to be central position within this coexpression network, which implied that they might exert important effects in HCC cuproptosis. Therefore, we chose the three lncRNAs for further experimentation Firstly, we designed a special smart silencer to knockdown the expression of NRAV, WARS2-AS1 and MKLN1-AS in two HCC cell lines. As shown in **Figures 11A–C**, the downregulation efficiency was verified by RT-qPCR. Then, a cell viability assay indicated that depletion of WARS2-AS1 and MKLN1-AS sensitized HCC cell to elesclomol-induced death in a dose-dependent manner, while NRAV deficiency had no effect on elesclomol (**Figures 11D–I**). Collectively, these results suggested that cuproptosis-related lncRNA WARS2-AS1 and MKLN1-AS played vital roles in HCC progression.

## Discussion

Cuproptosis, an uncharacterized cell death, which was proposed in 2022, has attracted researchers worldwide (10). However, the role of cuproptosis in cancer remains unknown, and greater effort is needed to reveal the underlying mechanisms of cuproptosis. In previous studies, lncRNAs have been reported to be closely connected with programmed cell death (PCD) including apoptosis, autophagy, necroptosis, and ferroptosis (15). For instance, overexpressed maternally expressed 3 (MEG3) was able to inhibit growth of breast cancer xenografts and promote cell apoptosis *via* regulating apoptosis related factors (32). Several lncRNA types may also facilitate tumorigenesis and ferroptosis resistance by acting as competing endogenous RNAs (33). Additionally, lncRNAs were able to protect tumor cells against necroptosis by blocking certain associated proteins (34). LncRNAs also had the ability to initiate autophagy by activating enzymes that were involved in the process (35). Furthermore, predictions of tumor patient outcome using lncRNA-associated signatures have been made using differentially expressed lncRNAs (36, 37). Currently, few research has been conducted to investigate the involvement of lncRNAs in cancer cuproptosis. Therefore, the discovery of CRLs is critical for the development of novel cancer targets. Here, we established a cuproptosis-related lncRNA signature for the first time, which was not only helpful for predicting prognosis and immune response in HCC patients but also presented a potentially effective strategy for guiding individual treatment.

In this study, CRG-associated lncRNAs from TCGA HCC RNA-seq data were initially analyzed followed by filtering out *via* differential analysis. Then, 221 CRDELs were subsequent to univariate cox regression analysis, and a total of 61 prognostic CRDELs were finally identified. Based on the 61 prognostic CRDELs, a lncRNA-CRG coexpression network was established. We observed a strong connection between LIPT1 and 29 prognostic CRDELs. LIPT1 is essential to lipoic acid metabolism, while lipoic acid is required for catalysis by multiple mitochondrial 2-ketoacid dehydrogenase complexes like pyruvate dehydrogenase (PDH) (38). Regulation of mitochondrial metabolism *via* PDH complex played a vital role in the process of cuproptosis (10). Meanwhile, we noticed that ATP7A was linked to 17 prognostic CRDELs. ATP7A acts as a Cu exporter to maintain intracellular Cu homeostasis. Once the balance is disrupted, it may affect multiple metabolic pathways and result in the occurrence of physiological disorders (39). Additionally, WARS2-AS1, ZNF32-AS2 and AC099850.4 were all co-expressed with four CRGs. Although the roles of WARS2-AS1 and ZNF32-AS2 have not been clarified previously, AC099850 was proved to be a tumor-promoting lncRNA to promote proliferation and invasion of HCC cells (40). The mechanism by which these lncRNAs regulate cuproptosis deserved to be explored in the future.

LASSO Cox regression analysis was applied to build a five-lncRNA prognostic signature, including FOXD2-AS1, NRAV, MED8-AS1, WARS2-AS1 and MKLN1-AS. The formula was used to calculate the risk score for each patient, and the median value was used to separate the patients into high- and low-risk groups. The patients in high-risk groups had a worse overall survival rate than that in the low-risk groups. Moreover, the predictive signature was shown to be accurate indicated by an excellent prediction performance *via* the ROC curve. Intriguingly, clinicopathological factors may not perform as well in predicting the prognosis of HCC patients as reliably as the predictive signature. In addition, internal validation also verified the formula’s predictive ability. In order to further investigate the relationship between the five-lncRNA model and cuproptosis, we compared the CRGs expression levels and the IC50 value of Cu ionophore elesclomol within the two groups. Results demonstrated that more than half of CRGs were dysregulated between the high- and low- risk groups, and elesclomol sensitivity was possibly more obvious in the low-risk group.

Furthermore, we conducted GSEA analysis to explore the biological processes and pathways between the two risk groups. Multiple immune-related pathways were shown to be strongly associated with high-risk patients, such as the inflammatory response, IL6/JAK/STAT3 signaling, T/B cell receptor signaling pathway, and chemokine signaling pathway and natural killer cell mediated-cytotoxicity. Inflammation is a well-documented cancer characteristic that plays an important role in the onset and progression of cancerous lesions. A growing body of data supports the importance of local and systemic inflammation in the growth of tumors and the survival of cancer patients (41). It was reported that glioma initiating CD133(+) cells and Mφs/microglia cointeraction activated expression of B7-H4 *via* IL6-activated STAT3, thereby blocking effective T-cell immune responses within the microenvironment of gliomas (42). Chemokines are chemotactic cytokines which are crucial for guiding immune cell movement and subsequently delivering an efficient anti-tumor immune response; however, chemokines also play a role in the production and recruitment of immune cells that contribute to a tumorigenic milieu (43). Considering the current evidence, we may conclude that tumor immunity is strongly associated with cuproptosis-related lncRNAs in HCC. In addition to immune-related signaling, we found that oxidative phosphorylation and the citrate cycle TCA cycle were mainly enriched in the low-risk group. As verified in the work by P. Tsvetkov *et.al*, mitochondrial aerobic respiration was the critical biological process affected by elesclomol induction (10). Therefore, we can deduce that CRLs might exert a vital role in the cuproptosis of HCC. Furthermore, combining immunotherapy with targeting CRLs is expected to become an optional alternative in the treatment of HCC.

Since there has been no evidence of a direct association between cuproptosis and immune cell infiltration in HCC, we used CIBERSORT to quantify the percentage of 22 kinds of tumor-infiltrating immune cells in HCC. Immune tolerance was shown in the high-risk HCC patients, due to significantly higher amounts of follicular helper T cells, regulatory T lymphocytes (Tregs), neutrophil and M0 macrophages. Emerging research has indicated that immune evasion and resistance to treatment are mediated by the tumor microenvironment in HCC, which includes tumor-associated macrophages (TAMs), cancer-associated fibroblasts (CAFs), Tregs, and myeloid-derived suppressor cells (MDSCs) (44). Combined with GSEA results, glycolysis was mainly related to the high-risk group. Glycolysis is the primary energy source for cancer cells, and they reshape their microenvironment to take advantage of this abundant supply (45). Previous research has shown that tumor glycolysis and tumor immune evasion are interdependent. Increased tumor glycolysis hampered the immune system’s ability to eliminate tumor cells (46). To our surprise, HIF-1α-stabilizing long noncoding RNA (HISLA) as signal transducers between TAMs and tumor cells was able to promote cancer aerobic glycolysis, which in turn contributed to the activation of TAMs through lactate release (47). Furthermore, PDL1-induced depletion of follicular helper T cells resulted in impaired B cell activity, accelerating the development of advanced HCC (48). An earlier study indicated that an increase in chemotaxis factor production might recruit neutrophil to produce an immunosuppressive milieu, resulting in the development of HCC (49). The role of immunotherapy in the treatment of HCC has been an increasingly hot topic in recent years (50). We next investigated the connection between the predictive signature and different immune checkpoints. Surprisingly, the patients in the high-risk groups showed a significant rise in the expression of PD1 and PDL1, implying that several immune checkpoint blockades may be beneficial for these individuals. It is therefore possible that the cuproptosis-related lncRNAs may be used to identify patients who are more likely to respond to anti-tumor immunotherapies. Apart from that, most differentially expressed CRGs within two risk groups were co-expressed with immune checkpoints significantly, providing a direct link towards immune response and cuproptosis. In addition, the expression level of the five CRLs in our signature was verified *via* RT-qPCR assay in our own HCC samples and cell lines. The expression trend was consistent with the prediction of the above bioinformatics research. Finally, the cuproptosis induced by elesclomol was further confirmed in our study, and this cytotoxicity was dinstinct from other traditional cell death. Three vital lncRNAs (NRAV, WARS2-AS1 and MKLN1-AS) were chosen for subsequent analysis. Results implied that only WARS2-AS1 and MKLN1-AS could regulate HCC cuproptosis significantly.

The findings of our research, however, had several limitations. Firstly, there is a lack of clinical follow-up data in-house to prove the accuracy of our predictive model. Additionally, although HCC cuproptosis was related to WARS2-AS1 and MKLN1-AS alteration, the exact molecular mechanisms controlled by WARS2-AS1 and MKLN1-AS needed more-in-depth investigations in depth.

In conclusion, our signature-based risk model outperformed standard clinicopathological parameters in terms of predicting survival. Low-risk groups exhibited more sensitive towards elesclomol than the high-risk group. Moreover, enrichment analysis revealed that several critical pathways were found to be associated with cuproptosis and immune response between the two groups, offering us novel insights into the treatment of HCC patients.

## Data availability statement

The original contributions presented in the study are included in the article/**Supplementary Material**. Further inquiries can be directed to the corresponding authors.

## Ethics statement

The studies involving human participants were reviewed and approved by Institutional Review Board of SRRSH. The patients/participants provided their written informed consent to participate in this study.

## Author contributions

HL and XF conceived and designed the experiments. DL, SJ, PC, YZ, YL, and CZ collected the data and performed the analysis. DL and SJ wrote the manuscript. All authors have read and approved the final manuscript.

## Supplementary material

Supplementary Figure 1 Differential expression analysis of lncRNAs in HCC samples compared to the normal tissues. Red indicated upregulated lncRNAs; Bule indicated downregulated lncRNAs; Grey indicated lncRNAs with no alteration. lncRNAs, long noncoding RNAs; HCC, hepatocellular carcinoma. *p < 0.05, **p < 0.01, and ***p < 0.001.

Supplementary Figure 2 Kaplan-Meier survival curves for high-risk and low-risk patient groups based on based on M stage, N stage, AFP, T stage and vascular invasion.

Supplementary Figure 3 The correlation of risk score and 22 types of TIICs in HCC using the CIBERSORT algorithm *via* Pearson test. TIICs, tumor-infiltrating immune cells; HCC, hepatocellular carcinoma.

Supplementary Figure 4 The correlation between differentially expressed CRGs within the two risk groups and immune checkpoints. CRGs, cuproptosis-related genes. *p < 0.05, **p < 0.01, and ***p < 0.001.
