# Supplementary material for: Comprehensive analysis of cuproptosis-related lncRNAs for prognostic significance and immune microenvironment characterization in hepatocellular carcinoma
Source: Front Immunol. 2023 Jan 4;13:991604. doi: 10.3389/fimmu.2022.991604 (PMC9846072; doi:10.3389/fimmu.2022.991604)
Supplement: Supplementary Figure 1 — Differential expression analysis of lncRNAs in HCC samples compared to the normal tissues. Red indicated upregulated lncRNAs; Bule indicated downregulated lncRNAs; Grey indicated lncRNAs with no alteration. lncRNAs, long noncoding RNAs; HCC, hepatocellular carcinoma. *p < 0.05, **p < 0.01, and ***p < 0.001. [file DataSheet_1.zip › supplementary figure.docx]

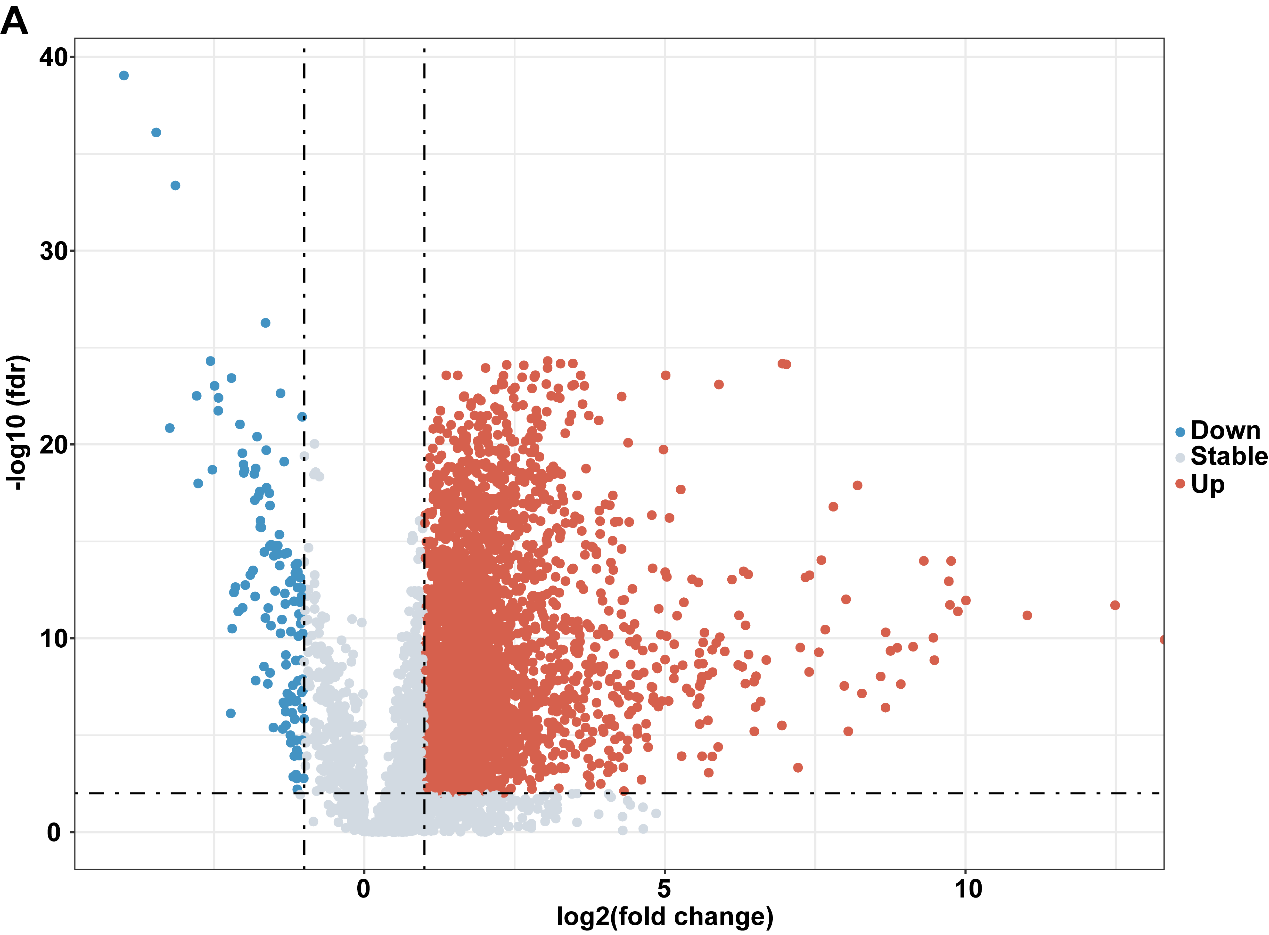


**Supplementary Figure 1**. Differential expression analysis of lncRNAs in HCC samples compared to the normal tissues. Red indicated upregulated lncRNAs; Bule indicated downregulated lncRNAs; Grey indicated lncRNAs with no alteration. lncRNAs, long noncoding RNAs; HCC, hepatocellular carcinoma. *p < 0.05, **p < 0.01, and ***p < 0.001.


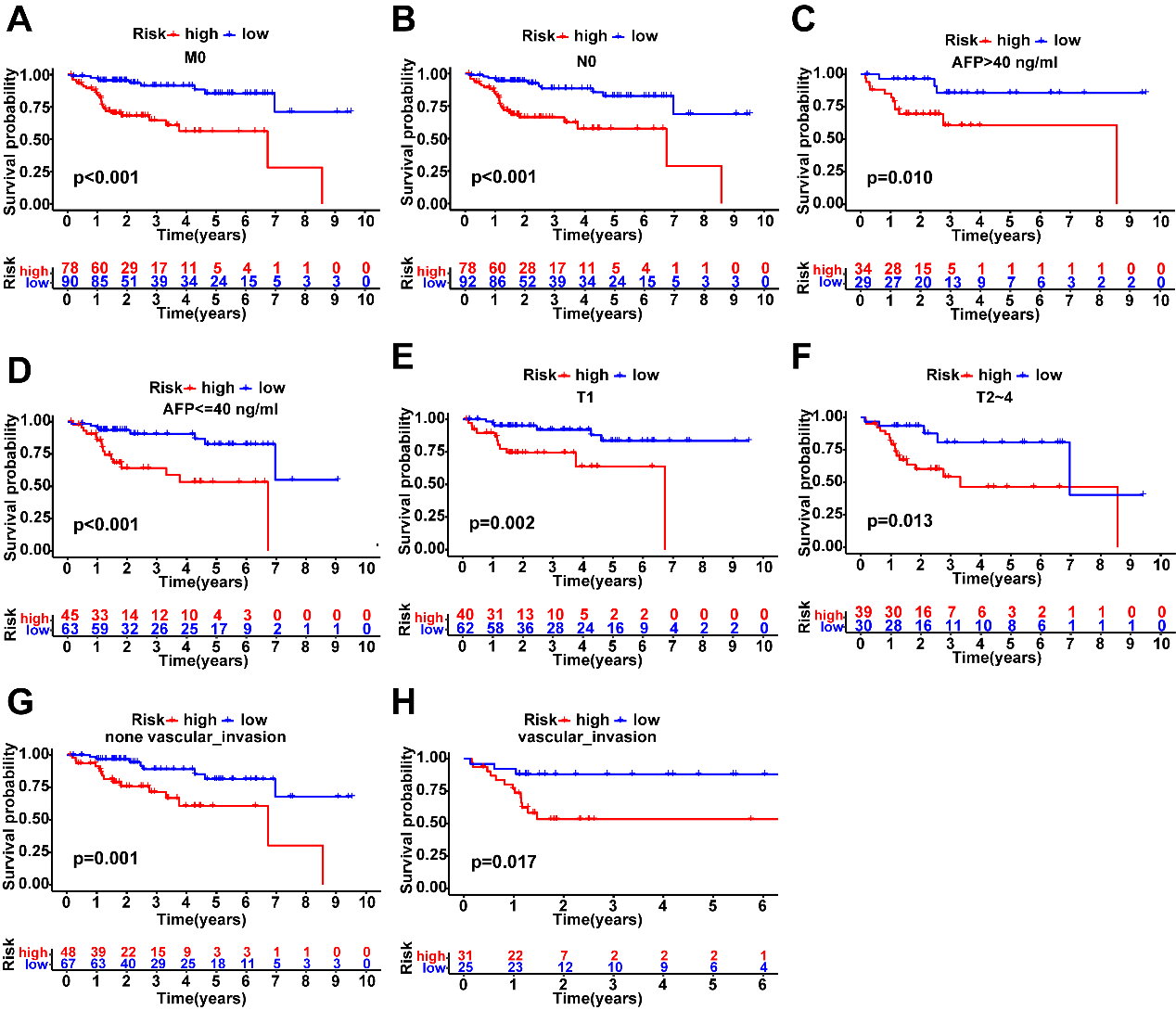


**Supplementary Figure 2**. Kaplan-Meier survival curves for high-risk and low-risk patient groups based on based on M stage, N stage, AFP, T stage and vascular invasion.


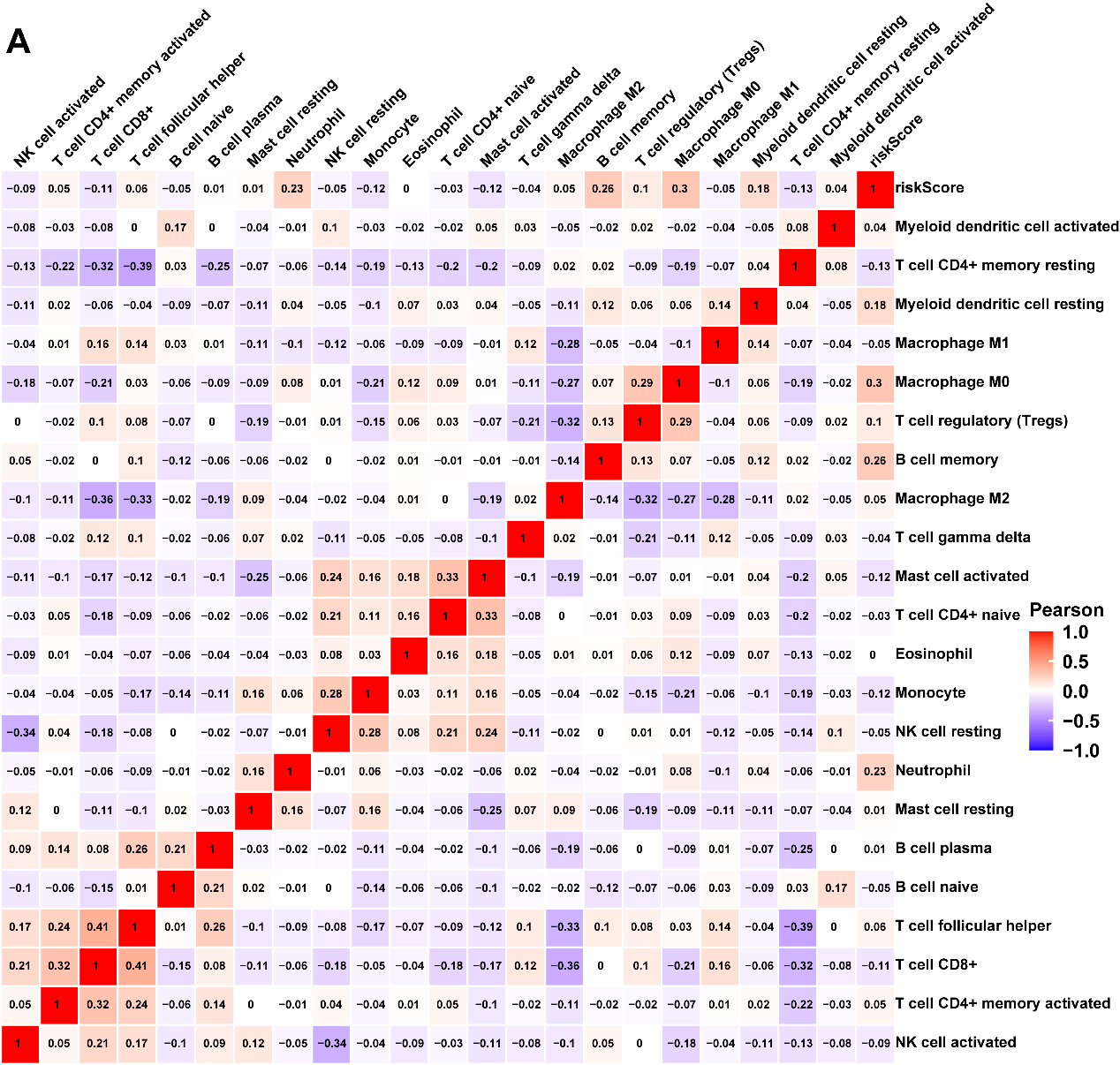


**Supplementary Figure 3**. The correlation of risk score and 22 types of TIICs in HCC using the CIBERSORT algorithm via Pearson test. TIICs, tumor-infiltrating immune cells; HCC, hepatocellular carcinoma.


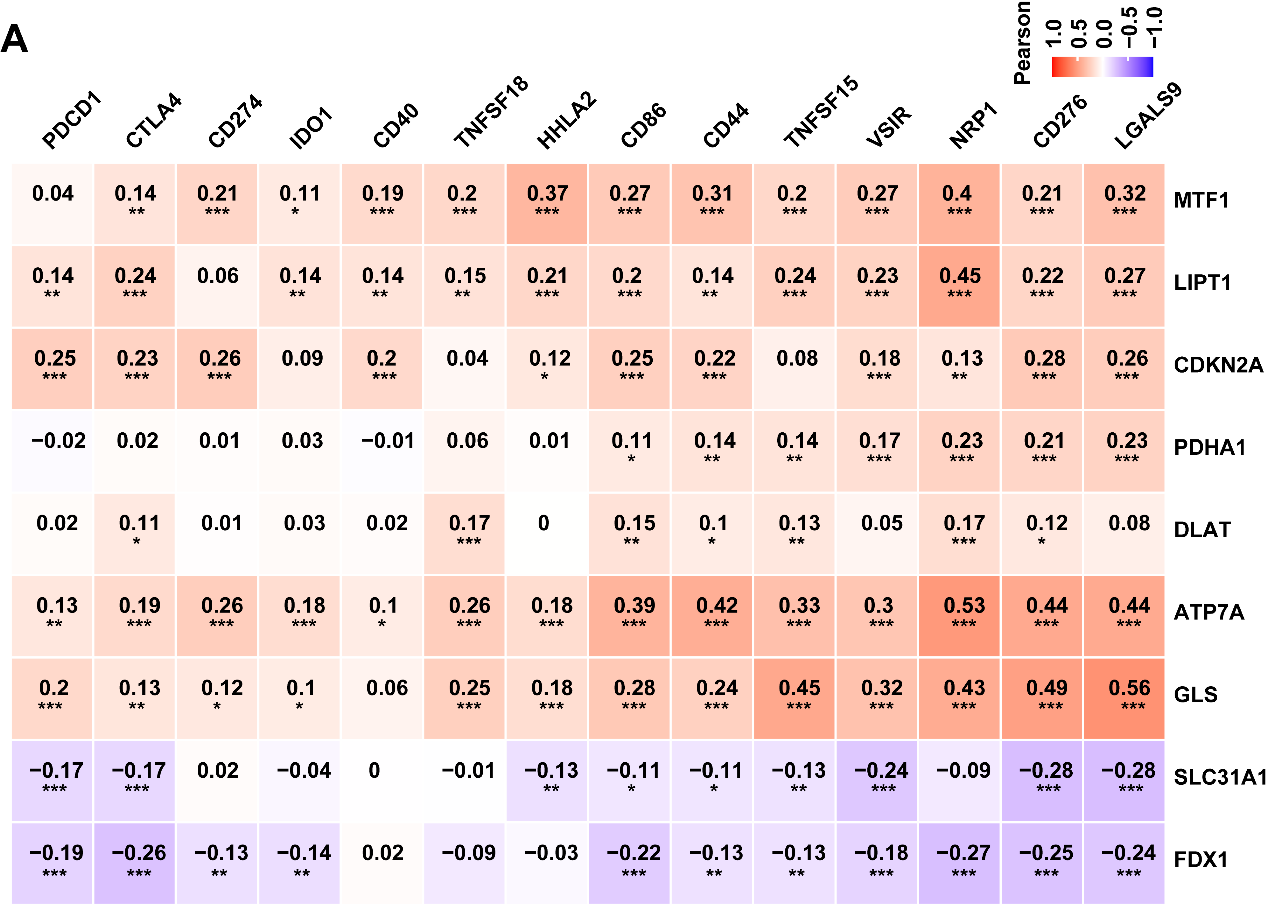


**Supplementary Figure 4**. The correlation between differentially expressed CRGs within the two risk groups and immune checkpoints. CRGs, cuproptosis-related genes. *p < 0.05, **p < 0.01, and ***p < 0.001.
