# Supplementary figures and images for: Comprehensive analysis of cuproptosis-related lncRNAs for prognostic significance and immune microenvironment characterization in hepatocellular carcinoma
Source: Front Immunol. 2023 Jan 4;13:991604. doi: 10.3389/fimmu.2022.991604 (PMC9846072; doi:10.3389/fimmu.2022.991604)

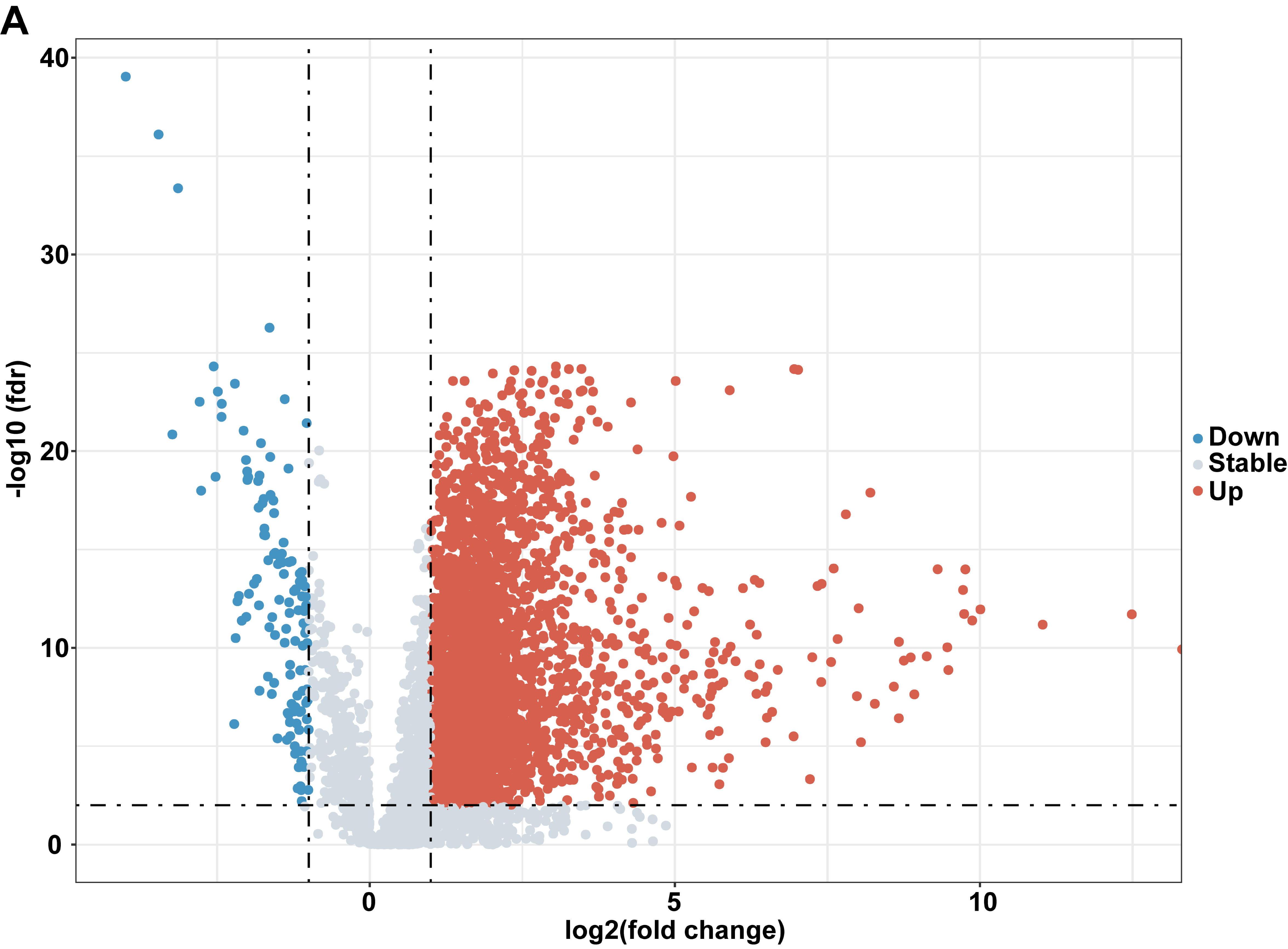

Supplement: Supplementary Figure 1 — Differential expression analysis of lncRNAs in HCC samples compared to the normal tissues. Red indicated upregulated lncRNAs; Bule indicated downregulated lncRNAs; Grey indicated lncRNAs with no alteration. lncRNAs, long noncoding RNAs; HCC, hepatocellular carcinoma. *p < 0.05, **p < 0.01, and ***p < 0.001. [file DataSheet_1.zip › supplementary Figure 1.tif]

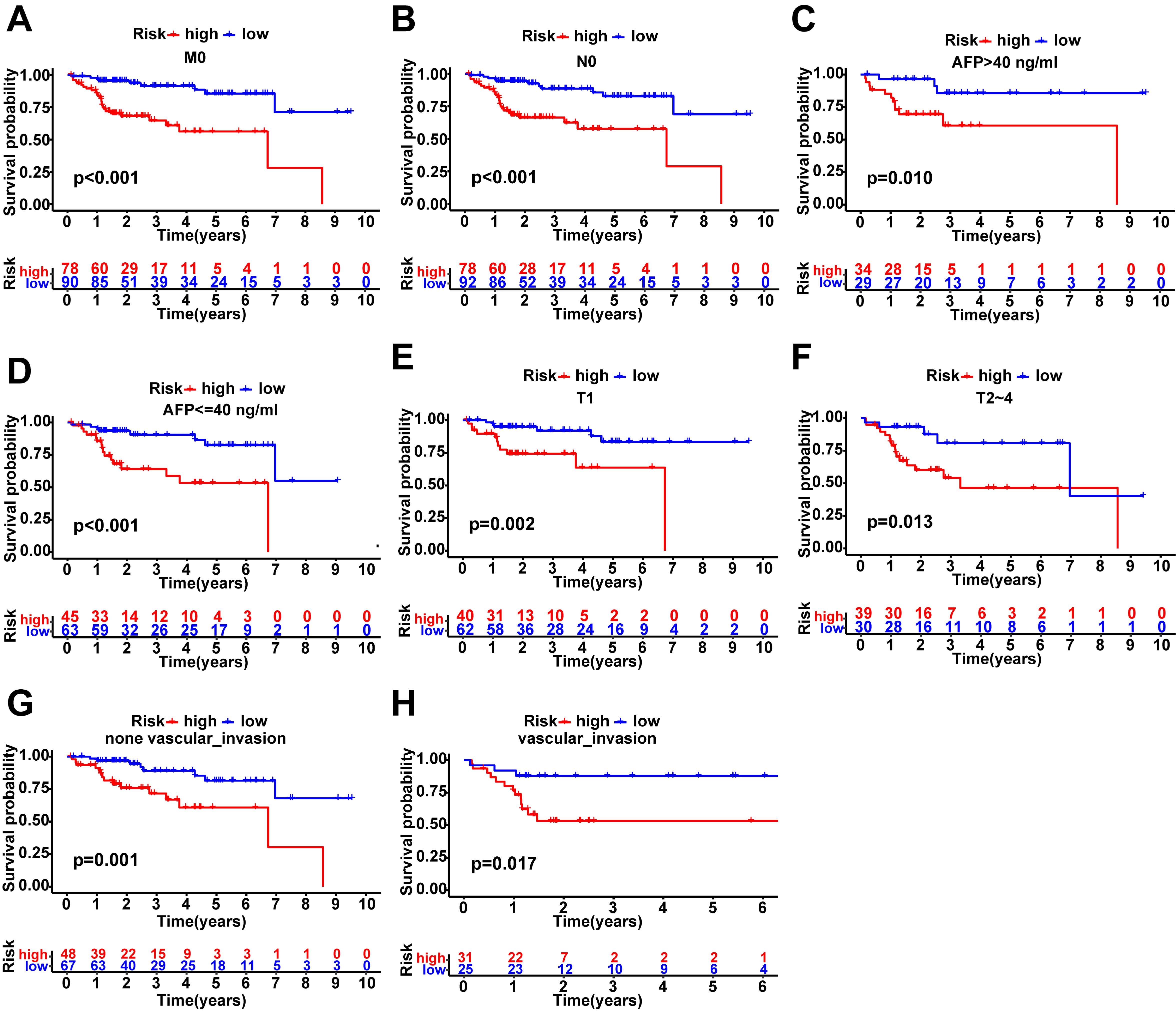

Supplement: Supplementary Figure 1 — Differential expression analysis of lncRNAs in HCC samples compared to the normal tissues. Red indicated upregulated lncRNAs; Bule indicated downregulated lncRNAs; Grey indicated lncRNAs with no alteration. lncRNAs, long noncoding RNAs; HCC, hepatocellular carcinoma. *p < 0.05, **p < 0.01, and ***p < 0.001. [file DataSheet_1.zip › supplementary Figure 2.tif]

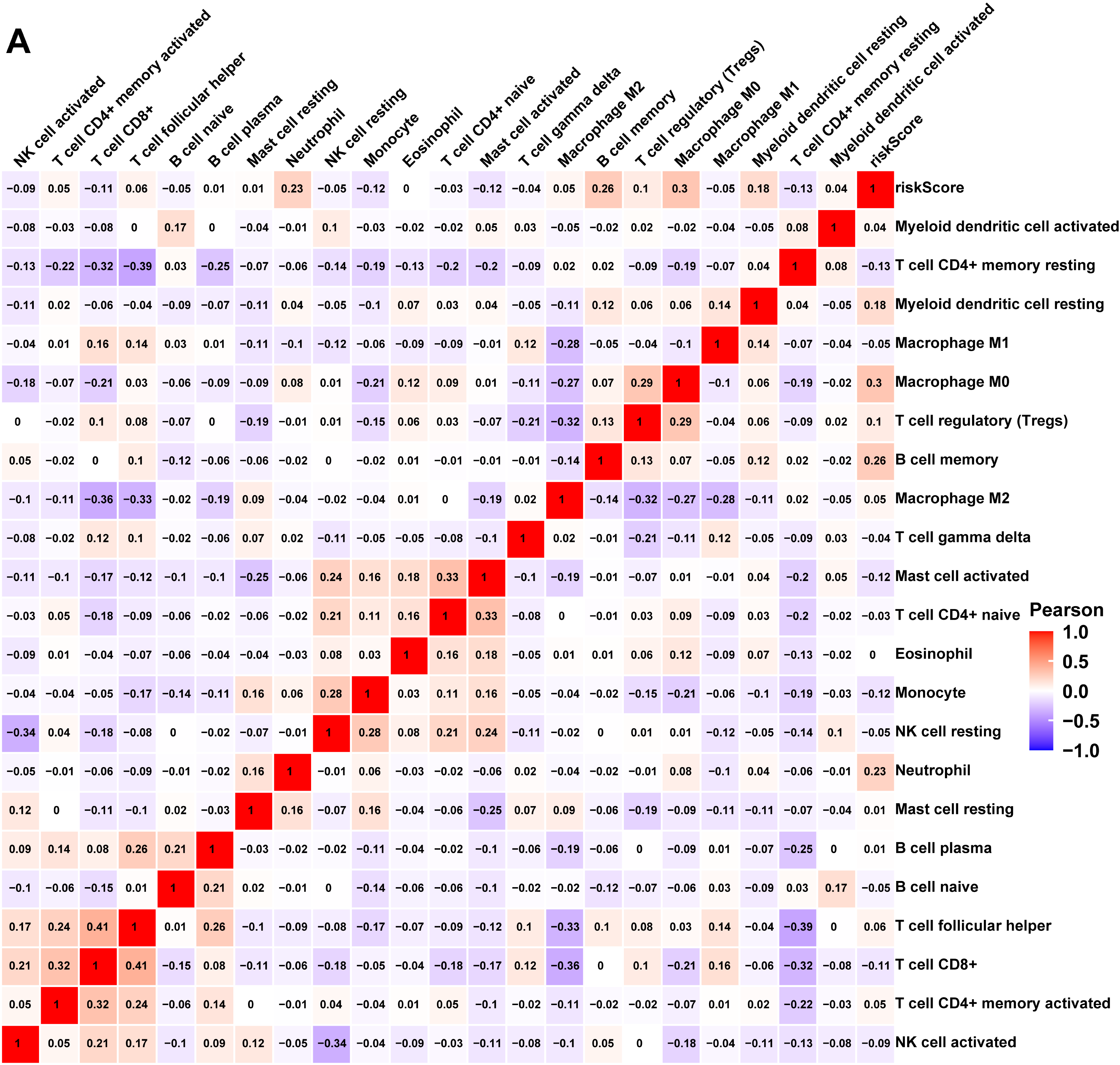

Supplement: Supplementary Figure 1 — Differential expression analysis of lncRNAs in HCC samples compared to the normal tissues. Red indicated upregulated lncRNAs; Bule indicated downregulated lncRNAs; Grey indicated lncRNAs with no alteration. lncRNAs, long noncoding RNAs; HCC, hepatocellular carcinoma. *p < 0.05, **p < 0.01, and ***p < 0.001. [file DataSheet_1.zip › supplementary Figure 3.tif]

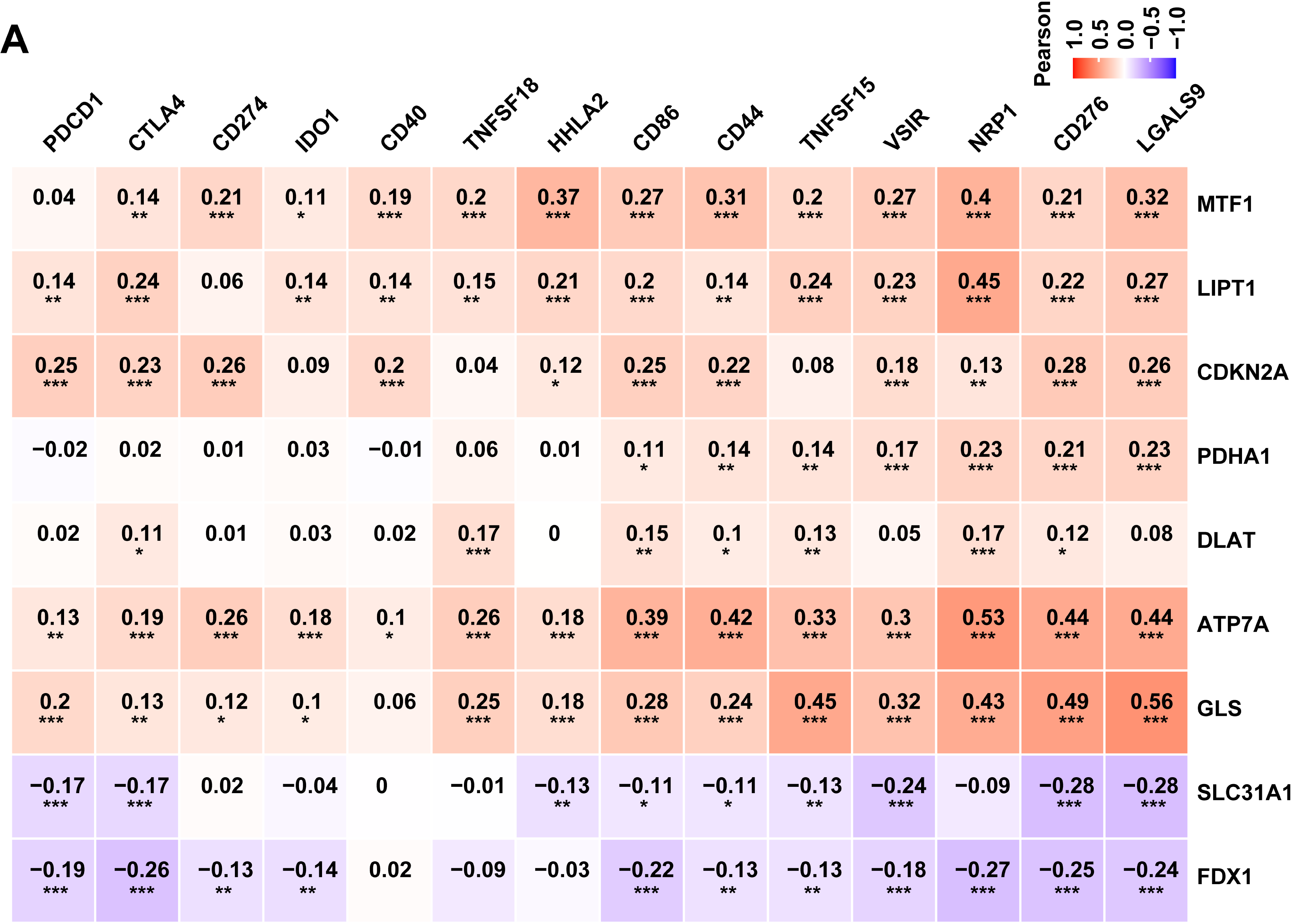

Supplement: Supplementary Figure 1 — Differential expression analysis of lncRNAs in HCC samples compared to the normal tissues. Red indicated upregulated lncRNAs; Bule indicated downregulated lncRNAs; Grey indicated lncRNAs with no alteration. lncRNAs, long noncoding RNAs; HCC, hepatocellular carcinoma. *p < 0.05, **p < 0.01, and ***p < 0.001. [file DataSheet_1.zip › supplementary Figure 4.tif]
